# The allosteric landscape of the Src kinase

**DOI:** 10.1126/sciadv.aea2726

**Published:** 2026-02-11

**Authors:** Antoni Beltran, Mohsin M. Naqvi, Andre J. Faure, Ben Lehner

**Affiliations:** ^1^ Centre for Genomic Regulation (CRG), Barcelona institute of Science and Technology, Carrer del Doctor Aiguader 88, Barcelona 08003, Spain.; ^2^ Institut de Biomedicina de València (IBV-CSIC), Carrer de Jaume Roig, 11, 46010 València, Spain.; ^3^ Wellcome Sanger Institute, Wellcome Genome Campus, Hinxton, UK.; ^4^ University Pompeu Fabra (UPF), 08002 Barcelona, Spain.; ^5^ Institució Catalana de Recerca i estudis Avançats (ICREA), 08010 Barcelona, Spain.

## Abstract

Enzymes catalyze the reactions of life and are the targets of many drugs. Most inhibitors bind conserved active sites, frequently lacking specificity. Targeting allosteric sites can increase specificity, reduce toxicity, and allow fine-tuning of activity; however, most allosteric sites in enzymes are unmapped. Here, we present a comprehensive experimental allosteric map of the Src protein kinase. We quantify the effects of more than 50,000 single and double amino acid substitutions on activity and abundance and use thermodynamic modeling to disentangle changes in fold stability and catalysis. The comprehensive energy landscape reveals that allostery across the kinase domain is extensive, directionally biased, and modulated by its regulatory domains. Inhibitory—but not activating—allosteric mutations show a strong distance-dependent decay away from the active site. Using the map, we identify multiple potentially druggable allosteric sites not previously reported in Src or other kinases. Our results establish a framework for comprehensive mapping of allostery in kinases and other enzymes important for medicine and biotechnology.

## Teaser

Comprehensive allosteric mapping prioritizes all possible druggable surface pockets in the Src kinase for allosteric inhibition.

## INTRODUCTION

Enzymes catalyze reactions important for nearly all biological processes, from gene expression to metabolism, signaling, and neuronal computation (*1*). Enzymes are also the largest class of targets of small-molecule therapeutics (*2*). Important examples include nucleotide metabolism and DNA replication enzymes in cancer and viral infections, lipid metabolism enzymes in metabolic disease, enzymes regulating synaptic transmission in neurological and psychiatric illnesses, and protein kinases in cancer and many other diseases (*2*, *3*). Most drugs targeting enzymes have an orthosteric mechanism of action, inhibiting activity by binding directly to a protein’s active site. However, the active sites of enzymes are typically highly conserved, making it difficult to specifically target one enzyme without also inhibiting many others from the same family. This is the case for protein kinases that catalyze the transfer of phosphate groups from adenosine 5′-triphosphate (ATP) to target proteins to regulate their activity. The human genome encodes 538 protein kinases, and orthosteric inhibitors targeting the ATP-binding site typically inhibit tens to hundreds of different kinases (*4*–*6*). The nonspecific inhibition of off-target enzymes by orthosteric drugs is a major cause of drug toxicity (*2*).

One strategy to increase drug specificity and reduce toxicity is to target allosteric sites (*7*). A key mechanism of enzyme control is allosteric regulation whereby catalytic activity is regulated by small molecule, metabolite, or macromolecule binding—or posttranslational modification—at a distant site (*8*). Such long-range allosteric transmission of information in proteins underlies nearly all biological regulation and has been deemed “the second secret of life” (*8*). Allosteric sites are less conserved between proteins, and allosteric drugs can therefore have higher specificity and reduced toxicity than orthosteric drugs (*7*). Targeting allosteric sites can also result in activation or in quantitative modulation of activity, for example, tuning the response to an agonist (*9*).

Most enzymes and most proteins, however, have no known allosteric sites to target therapeutically. The prevalence of allostery across the protein universe is unknown. A key reason for this is the lack of methods that systematically quantify allosteric communication (*10*–*12*). Fast and general methods to globally quantify allosteric regulation of enzyme activity would transform our ability to understand, predict, target, and engineer allosteric control.

Because of their pivotal role in many physiological and pathological processes, protein kinases now constitute the second most important class of small-molecule drug targets (*13*, *14*). In cancer, driver mutations hyperactivate kinases and kinase signaling, and effective kinase inhibitors have been developed (*13*, *14*), including inhibitors of BCR-ABL in chronic myeloid leukemia (*15*), BRAF and mitogen-activated protein kinase (MEK) inhibitors in melanoma (*16*), epidermal growth factor receptor (EGFR) and ALK inhibitors in lung cancer (*17*), and EGFR/HER2 inhibitors in breast cancer (*18*). Most kinase inhibitors developed to date have an orthosteric mechanism of action, targeting the highly conserved ATP-binding pocket, resulting in limited specificity (*13*, *14*). Moreover, the efficacy of many kinase inhibitors in cancer is short-lived because of selection for resistance mutations (*13*, *14*, *19*).

To increase specificity and overcome resistance mutations, allosteric inhibitors have been developed against several kinases (*20*). These include asciminib, a highly selective inhibitor targeting the myristate-binding site of BCR-ABL (*21*, *22*); trametinib and selumetinib, MEK1/2 inhibitors that bind a pocket located in the vicinity of the ATP-binding site and helix αC (*23*); and MK-2206, an AKT inhibitor that binds at the interface between its PH and kinase domains (KD) (*24*), among many others (*20*). However, although allostery is likely part of the regulation of all kinases (*25*), most kinases have no known allosteric sites (*26*). Rather, every kinase has tens of different surface pockets that could be potentially targeted with small molecules, and it is not known which of these pockets—if any—is allosterically active (*27*). In short, we do not know which pockets in which kinases are allosterically active and which should be prioritized for drug development.

Here, we present a general approach that can be used to globally quantify allosteric regulation of enzyme activities. We use the method to produce a comprehensive map of positive and negative allosteric control of an enzymatic activity, the Src kinase. *Src* was the first discovered oncogene (*28*) and is a notable drug target in cancer due to its role in regulating cell growth, cell migration, and angiogenesis (*29*). Using in vivo assays, we quantify the effects of more than 50,000 single and double mutant variants on the activity and abundance of the Src kinase for the KD in isolation and in the context of the full-length enzyme, totaling 30 separate pooled selection assays and more than 200,000 variant effect measurements. We use energy modeling to disambiguate the effects of mutations on kinase activity and abundance, thereby revealing complete allosteric maps of the Src KD alone and in the context of its regulatory domains. Allosteric control of Src is pervasive, distance dependent, anisotropic (direction dependent), and reasonably well predicted by sequence and structural features. These maps enable the comprehensive analysis of the positive and negative allosteric control of the druggable surface of an enzyme, allowing unbiased genetic prioritization for drug discovery of surface pockets not previously reported as allosteric in any kinases. We believe that this approach will allow comprehensive allosteric maps to be charted for many other kinases and therapeutically and biotechnologically important enzymes.

## RESULTS

### High-throughput mapping of allosteric regulation of enzyme activity

Most enzymes have no known allosteric sites to target for drug discovery or biotechnological control. We therefore conceived a general strategy that uses genetics to comprehensively quantify allosteric regulation of enzyme activity.

Our approach has three steps: First, the effects of mutations on catalytic activity are quantified; second, the effects of the same mutations on the abundance of the folded enzyme are measured; and third, changes in activity that cannot be accounted for by changes in concentration are quantified by fitting a model to the data. Because of the nonlinear relationships between the energetic effects of mutations and molecular phenotypes, we quantify the effects of mutations in multiple starting protein variants with different abundance and/or activities to constrain model fitting, allowing the underlying causal effects of mutations to be determined (*12*, *30*–*32*). Both enzymatic activity (*33*–*36*) and protein abundance (*37*–*41*) can be quantified using many different selection assays. Thus, although, here, we demonstrate the approach using a selection assay suitable for one particular class of enzymes (protein kinases) and one particular method to quantify folded protein abundance, this general approach can be used to quantify allosteric regulation in any enzyme, provided that both activity and folded abundance can be quantified at scale.

### Measuring activity and abundance of Src protein kinase variants at scale

To demonstrate the feasibility of the approach, we apply it here to the human oncoprotein Src. Src was the first discovered tyrosine kinase and the first identified, cloned, and sequenced oncogene (*28*, *42*). We measured the enzymatic activity of Src variants using a highly validated cellular toxicity assay where the inhibition of yeast growth is directly proportional to the levels of Src-induced protein phosphorylation (*43*–*45*). The abundance of folded Src can also be quantified in the same yeast cells using the sandwich abundance protein-fragment complementation assay(sPCA). sPCA uses protein fragment complementation between two dihydrofolate reductase (DHFR) fragments fused at both termini of Src to quantify soluble protein concentration over at least three orders of magnitude (*12*, *31*, *41*, *46*).

**Fig. 1.**
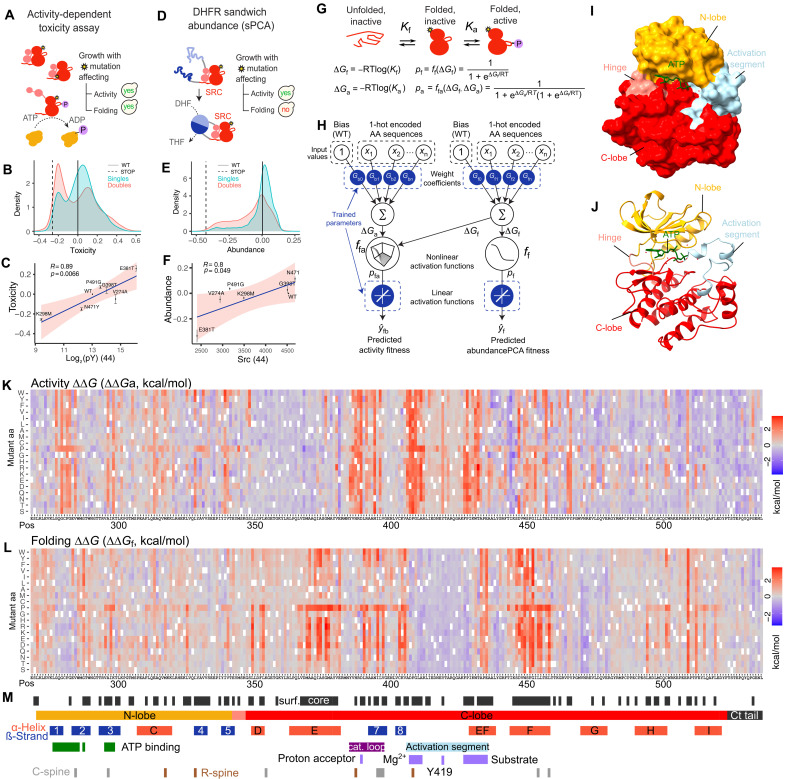
Mapping the energetic and allosteric landscapes of enzymes. (**A**) Overview of the toxicity selection assay to measure the protein kinase activity of Src KD variants at scale. yes, yeast growth; no, yeast growth defect. (**B**) Activity fitness distribution of single and double mutants of Src. (**C**) Correlation of activity fitness measurements to in vivo phosphotyrosine levels (*44*). (**D**) Overview of the sandwichPCA (sPCA) selection assay to measure in vivo abundance of Src variants at scale. DHF, dihydrofolate; THF, tetrahydrofolate. (E) sPCA fitness distribution of single and double mutants of Src. (**F**) Correlation of sPCA fitness measurements to in vivo Src abundance (*44*). (**G**) Three-state equilibrium and corresponding thermodynamic model. ∆G_f_, Gibbs free energy of folding; ∆*G*_a_, Gibbs free energy of the active state; *K*_f_, folding equilibrium constant; *K*_a_, inactive-active state equilibrium constant; pf, fraction folded; pfa, fraction folded and active; ff, nonlinear function of ∆*G*_f_; ffa, nonlinear function of ∆G_f_ and ∆G_a_; *R*, gas constant; *T*, temperature in Kelvin. (**H**) Neural network architecture used to fit thermodynamic models to the toxicity and abundance data (bottom, target and output data), thereby inferring the causal changes in free energy of folding and binding associated with single amino acid substitutions (top, input values). (**I** and **J**). Three-dimensional structure of the Src KD [Protein Data Bank (PDB) ID: 2SRC]. (**K** and **L**). Heatmaps showing inferred changes in activity free energies [(K) ∆∆*G*_a_] and folding free energies [(L) ∆∆*G*_f_]. (**M**) Sequence and annotation of Src. Locations of individual secondary structure elements and functional regions were obtained from (*50*, *74*).

To generate sufficient data for model fitting, we constructed five overlapping libraries of Src variants that together cover all possible single mutants in the KD (see sequences in table S6), with each variant present in at least 10 different genetic backgrounds (table S7). The genetic backgrounds were selected to provide a range of different Src activities due to either abundance or catalytic activity changes (see Materials and Methods). In total, the five libraries contain a total of 54,455 genotypes. We quantified the kinase activity and abundance of each genotype in 30 separate pooled selection assays. First, we quantified kinase activity in three biological replicates using kinase-dependent impairment of cell growth (Fig. 1, A and B) (*43*–*45*, *47*). Activity scores were highly reproducible for all five library tiles (median Pearson’s *r* = 0.93) and strongly correlated with Src-dependent tyrosine phosphorylation (*43*, *44*) (*r* = −0.89; Fig. 1C and fig. S1, A and B). Second, we quantified the cellular abundance of Src KD variants using sPCA (Fig. 1, D and E) (*31*, *41*) in three biological replicates. Abundance measurements were also reproducible (median *r* = 0.93; fig. S1, A and C) and correlated with the in vivo abundance of SRC variants measured by Western blotting (*r* = 0.80; Fig. 1F) (*43*, *44*). Measured fitness scores for all genotypes and their associated errors are provided in table S1.

### Quantifying changes in activity not caused by changes in abundance

To quantify the changes in Src activity that are not accounted for by changes in the cellular abundance of Src, we fit a simple phenomenological “enzyme folding and activation” model to the data (see Materials and Methods). In this model, the folding of Src is explicitly modeled using a two-state thermodynamic model in which the protein can exist in two states—unfolded (U) and folded (F)—with the Gibbs free energy of folding, ∆*G*_f_, determining the partitioning of Src molecules between the two states according to the Boltzmann distribution (Fig. 1, G to M) (*12*, *31*, *32*). All other biophysical changes that alter Src kinase activity are quantified using a second pseudo–free energy, which we refer to as the activity energy, ∆*G*_a_ (Fig. 1, G to M). The model is formally equivalent to a three-state model with unfolded (U), folded inactive (F), and folded active (A) states. The active state modeled here is phenomenological and designed to quantify all changes in activity not accounted for by changes in total soluble protein abundance. Although shifts in the equilibrium between inactive and active kinase conformations will be captured as changes in ∆G_a_, so too will other molecular mechanisms that affect catalytic activity (*k*_cat_) and substrate affinity (*K*_m_) independently of the conformational state of Src.

Fitting the enzyme folding and activation model to the data provides very good prediction of the abundance and activity of double mutants (median percent variance explained 72.3% for activity and 84.1% for abundance, evaluated by 10-fold cross validation; fig. S1D) and a marked improvement over a two-state (unfolded and folded active) model (fig. S1E). In contrast, increasing the complexity of the model to four states did not improve performance (fig. S1E). In total, our dataset quantifies folding and activity energies for all of the 5111 possible single substitution variants in the Src KD (table S2).

### Stability of the kinase fold

**Fig. 2.**
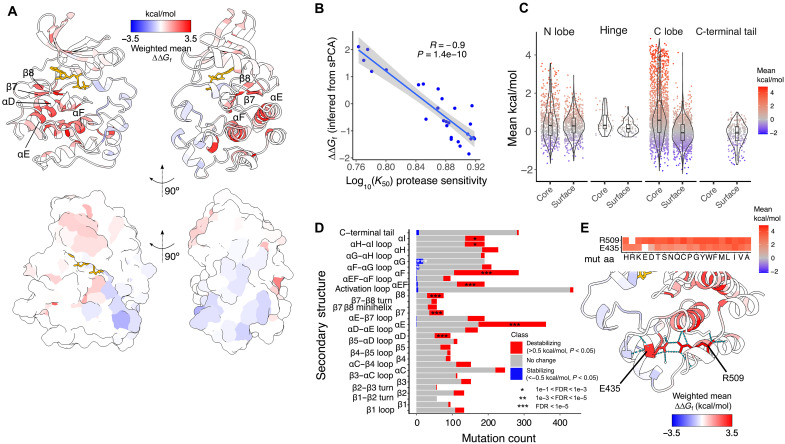
The folding landscape of the Src KD. (**A**) Structure of the Src KD colored by the per-site weighted mean ∆∆*G*_f_ (PDB ID: 2SRC). The secondary structure elements most enriched in destabilizing mutations are annotated. (**B**) Correlation between trypsin sensitivity measured as log_10_(*K*_50_) and ∆∆*G*_f_ inferred from the sPCA data for full-length Src using thermodynamic modeling. (**C**) ∆∆*G*_f_ distributions across the core and surface of the two kinase lobes. (**D**) Enrichment of destabilizing and stabilizing mutations in secondary structure elements of Src (FET). * 1e-1 < FDR < 1e-3, ** 1e-3 < FDR < 1e-5, *** FDR < 1e-5. (**E**) ∆∆*G*_f_ of mutations in the E435-R509 salt bridge. aa, amino acid.

Our data provide comprehensive measurements of how mutations affect the in vivo abundance of the protein kinase fold (Fig. 2A and movie S1). To validate the inferred folding energies, we used mRNA display coupled to in vitro proteolysis sensitivity (*39*) where in vitro–transcribed and translated Src KD variants are treated with a gradient of trypsin concentrations, with unfolded variants displaying increased accessibility to trypsin digestion. *K*_50_ values indicating the concentration of trypsin resulting in half maximal cleavage rate measured using this method correlated well with ∆∆*G*_f_ inferred from sPCA measurements (Pearson’s *r* = −0.90, *P* = 3.5 × 10^−8^, *n* = 27 variants; Fig. 2B).

The Src KD is composed of two structurally and functionally distinct subdomains—the N and C lobes—with the active site located in the cleft between the two. The N lobe is mostly composed of β strands and contains the ATP-binding site, whereas the C lobe is mostly α-helical and ends in a disordered C-terminal tail that regulates the conformational state of the kinase (*48*–*50*). Mutations have a wide range of effects, with many destabilizing [1142 with ∆∆*G*_f_ > 0.5 kcal/mol, false discovery rate (FDR) < 0.1, *z* test] and a small number of stabilizing variants (48 with ∆∆*G*_f_ < −0.5 kcal/mol, FDR < 0.1, *z* test).

Destabilizing mutations across the KD show strong structural biases—with core mutations (relative solvent accessible surface area, rSASA <0.25) being much more likely to be destabilizing than mutations on the surface [odds ratio (OR) = 6.30, *P* = 8.73 × 10^−132^, Fisher’s exact test (FET); Fig. 2, A and C]. This enrichment is much stronger for the C lobe (OR = 7.49, *P* = 4.42 × 10^−108^, FET) than for the N lobe (OR = 2.47, *P* = 8.77 × 10^−9^, FET), with mutations in the C-terminal tail having very mild effects on stability (Fig. 2C). The hydrophobic αF helix buried in the C-lobe core is most critical for stability (Fig. 2D), along with the αE helix that contacts αF extensively, αEF, and β7 and β8 that are packed against αE (Fig. 2, A and D). Together, these elements form the main stabilizing core of the kinase fold (Fig. 2, A and D). Mutations to proline are most likely to be destabilizing, especially in helices and β strands (Fig. 1L and fig. S2A) (*51*). Of all long-range side-chain to side-chain structural contacts, the salt bridge connecting R509 in helix αI with E435 in αEF is the most sensitive to mutation (maximum mean ∆∆*G*_f_ of residue pairs), with no substitutions tolerated in either residue (Fig. 2E). Mutations in E435-R509 are 2.63-fold more destabilizing than mutations in the next most sensitive contact (the E342-K404 salt bridge), 3.19-fold more than the average of all salt bridges, and 3.85-fold more than the average of all side-chain to side-chain contacts. The two structurally distinct lobes of the Src KD thus contribute differentially to the in vivo stability of the domain, with the more dynamic ATP-binding N lobe displaying a higher tolerance to mutagenesis than the larger and more compact C lobe. 

Quantifying mutational effects across a range of destabilized genetic backgrounds allows us to identify a total of 48 mutations that increase the cellular abundance of the Src KD, which we define as in vivo stabilizing variants (∆∆*G*_f_ <−0.5, FDR < 0.1, *z* test; Fig. 2D), distributed across 25 positions in the Src KD. A total of 27 of 48 stabilizing mutations map to a cluster formed by the alphaG helix (OR = 18.70, FDR = 4.01 × 10^−14^) and in the alphaF-alphaG loop (OR = 4.83, FDR = 1.01 × 10^−2^) on the KD surface.

### Molecular origins of loss of function in the Src kinase

**Fig. 3.**
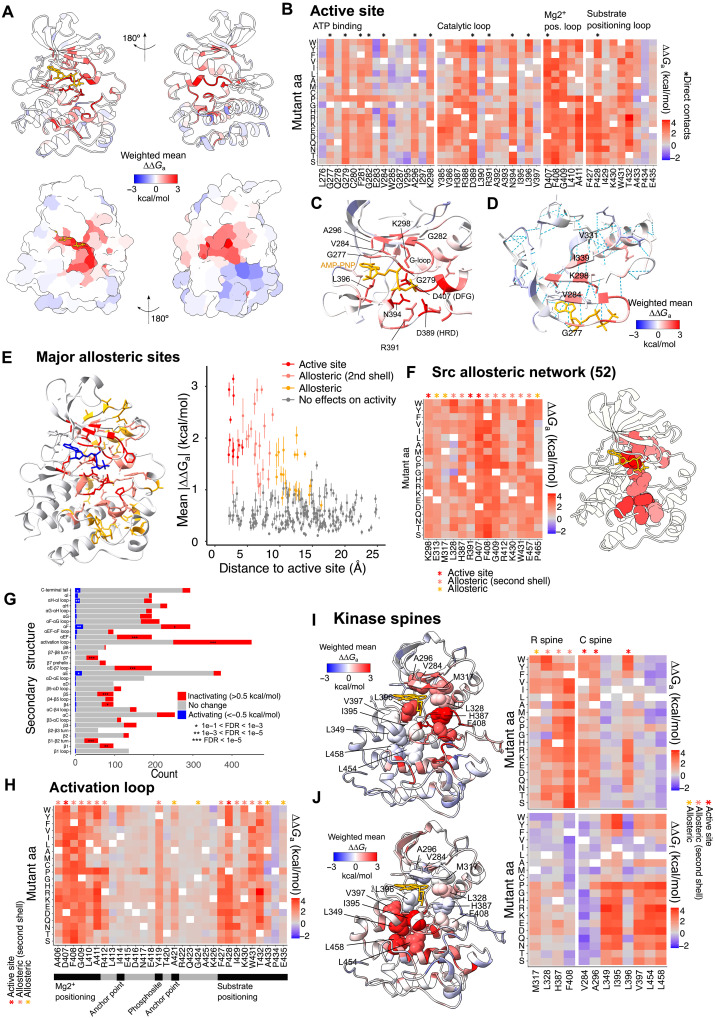
The allosteric landscape of the Src KD. (**A**) Structure of the Src KD colored by the per-site weighted mean ∆∆*G*_a_ (PDB ID: 2SRC). (**B**) Heatmap showing ∆∆*G*_a_ of residues in the Src regions forming the active site. Residues directly contacting the nucleotide, Mg^2+^ or the substrate peptide are labeled with stars. (**C**) The active site of Src (PDB ID: 2SRC). (**D**) β Sheet forming the top surface of the ATP-binding pocket (N lobe). (**E**) Relationship between per site–averaged ∆∆*G*_a_ and the minimum heavy atom distance to the active site. Error bars represent the SEM. (**F**) Heatmap showing ∆∆*G*_a_ of mutations in residues that are part of a previously described allosteric network connecting ATP and substrate binding sites (*52*). (**G**) Enrichment of inactivating and activating mutations in secondary structure elements (FET). (**H** and **I**) Src structures and heatmaps showing ∆∆*G*_a_ of mutations in the catalytic (C) and regulatory (R) spines of Src. (**J**) Src structures and heatmaps showing ∆∆*Gf* of mutations in the catalytic (C) and regulatory (R) spines of Src.

Our data provide a complete map of the effects of mutations on protein kinase activity independently of their effects on protein abundance (Fig. 3A and movie S2). In total, we identified 1070 mutations that modulate kinase activity more than can be accounted for by changes in Src abundance (|∆∆G_a_| > 0.5, FDR < 0.1, *z* test). Nine hundred and sixty-one of these mutations are inhibitory, and 109 are activating. Of all 1893 unique loss-of-function mutations in Src, 883 are only destabilizing, 751 are only inactivating, and 259 affect both phenotypes (fig. S3). Loss of function via protein destabilization in the Src kinase fold is thus marginally more likely than specific loss of activity.

### The Src active site

Mutations in the active site—residues that directly contact ATP, Mg2^+^, or the substrate peptide phosphosite—are overwhelmingly detrimental to kinase activity, with 225 of 247 decreasing activity (∆∆*G*_a_ > 0.5, OR = 30.04, *P* = 5.21 × 10^−119^, FET; Fig. 3, B and C). Inactivating variants are strongly enriched in the ATP-binding site (OR = 3.42, *P* = 2.35 × 10^−19^, FET), the catalytic loop (HRD motif, OR = 5.78, *P* = 1.01 × 10^−7^, FET), the Mg2^+^ positioning loop (DFG motif, OR = 51.49, *P* = 7.27 × 10^−55^, FET), and the substrate positioning loop (OR = 6.84, *P* = 1.26× 10^−32^, FET) (Fig. 3B). Mutations in the β sheet that forms the top surface of the ATP-binding pocket have a notable alternating pattern of mutational effects, with substitutions of side chains pointing toward the nucleotide detrimental for activity (Fig. 3D).

### Major allosteric sites

We identified 861 mutations in 168 sites located outside of the active site that modulate kinase activity (|∆∆Ga| > 0.5, FDR < 0.1, *z* test). A total of 752 of these allosteric mutations are inhibitory, and 109 activate the kinase. We define major allosteric sites as residues outside the active site that are enriched in mutations modulating kinase activity (Fig. 3, E and F). By this definition, Src has 42 major allosteric sites (OR > 2, FDR < 0.1, FET): 7 in the N lobe and 35 in the C lobe (movie S3).

Strikingly, 21 of 42 major allosteric sites are second shell sites directly contacting residues in the active site. The major allosteric sites also include all 11 nonactive site residues previously predicted to be part of an allosteric network that communicates between the substrate and ATP-binding sites of Src (Fig. 3F). This network was predicted via analysis of changes in electrostatic and hydrophobic contacts between active and inactive conformations in molecular dynamics simulations (*52*). Of these 11 previously predicted allosteric positions, 8 are second shell residues. The predicted allosteric network is very strongly enriched for mutations with large ∆∆*G*_a_ (OR = 14.57, *P* = 2.10 × 10^−71^, FET, excluding active site residues, and OR = 7.54, *P* = 2.00 × 10^−10^, FET, excluding active site and second shell residues), with all individual residues enriched at least sixfold for allosteric mutations (Fig. 3F).

### Inhibitory allosteric mutations

Defining major inhibitory allosteric sites as sites enriched for inhibitory mutations (OR > 2, FDR < 0.1, FET) identifies 44 positions, 42 of which are also major allosteric sites. Inhibitory allosteric mutations are enriched in several structural elements (Fig. 3G), including helix αC, consistent with its conformational change upon Src activation (*48*, *50*). Inhibitory mutations are concentrated in residues on the inner surface of helix αC, including E313 that engages in a salt bridge with K298 in the active state (Fig. 3F), the R-spine (see below) residue M317, and the hydrophobic residues F310, A314, and L320. The β4 and β5 strands located between αC and the active site are also enriched for allosteric mutations, but to a lesser extent (Fig. 3G). Inhibitory allosteric mutations are also abundant in the activation loop (Fig. 3, G and H) including in Y419, which locks Src in the active state when phosphorylated (Fig. 3H) (*50*, *53*); in αEF, which functions in substrate positioning; and in αG, in hydrophobic residues that contact αEF.

Last, inhibitory allosteric mutations are enriched in the αF helix that acts as an anchor for the catalytic (C) and regulatory (R) “spines” (Fig. 3, H and I). The C and R spines are two groups of residues that are not contiguous in the primary sequence of kinases but form a bipartite hydrophobic core in catalytically active kinases (*54*, *55*). Mutations in all R-spine residues have strong inactivating effects independently of their effects on abundance (Fig. 3H). In contrast, only the C-spine residues in direct contact with ATP (A296, V284, and L396) are enriched in inactivating mutations (Fig. 3H). Mutations in the rest of the C-spine sites have small effects on ∆*G*_a_, and their strong effects on kinase activity at the fitness level are almost fully explained by a loss of fold stability (Fig. 3I).

### Activating allosteric mutations

In total, 11 residues outside of the active site are enriched for activating mutations, which we define as major activating allosteric sites (OR > 1, *P* < 0.05, FET; fig. S4), all of which are located in the C lobe. Many of these sites map to the αF pocket (E381, T443, I444, K445, and P506), a previously defined hotspot of activating mutations (*44*). Last, activating mutations are also enriched in Y530, the phosphosite in the C-terminal tail that mediates Src autoinhibition and locks the kinase in a closed conformation when phosphorylated (*48*–*50*).

### The distance dependence of allosteric regulation

**Fig. 4.**
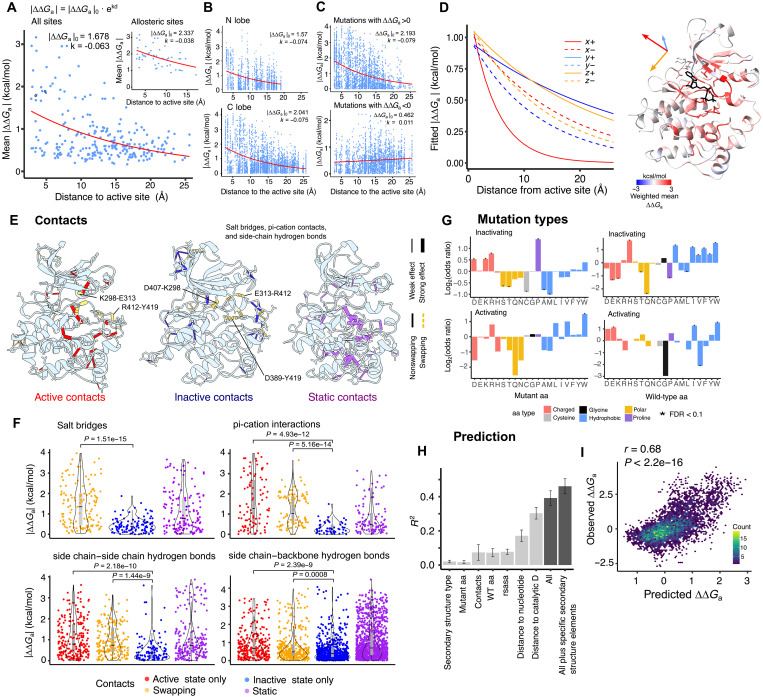
Allosteric communication is anisotropic and enriched in dynamic structural contacts. (**A**) Exponential decay fits to the relationship between per site–averaged |∆∆*G*_a_| to the minimum heavy atom distance to the active site, for all sites (main) and for active and allosteric sites (inset). |∆∆*G*_a_|_0_ = starting ∆∆*G*_a_ at distance = 0 (active site), *k* = decay rate, *d* = distance from the active site. (**B**) Exponential decay of |∆∆*G*_a_| with distance for the N and C lobes. (**C**) Exponential decay of |∆∆*G*_a_| with distance for inactivating (∆∆*G*_a_ > 0) and activating (∆∆*G*_a_ < 0) mutations. (**D**) Exponential decays in the three orthogonal spatial directions, positive and negative. Decay was calculated in each spatial direction for residues located at a distance less than 10 Å from the active site in the two remaining directions. (**E**) Structures depicting noncovalent contacts specific to the active state of Src (PDB ID: 1Y57), inactive state of Src (PDB ID: 2SRC), and present in both. Salt bridges, side chain–side chain and side chain–backbone hydrogen bonds, and pi-cation contacts are shown. Thickness is proportional to the averaged ∆∆G_a_ of mutations in contacting residues. Contacts in residues that swap partners between active and inactive conformations (“swapping residues”) are depicted as yellow dashed lines. (**F**) Distributions of ∆∆*G*_a_ of mutations in residues classified according to their contacting patterns, for four contact types. Adjusted *P* values of Wilcoxon rank sum tests are shown. (**G**) Enrichments of activating and inactivating mutations according to WT and mutated amino acid identities. (**H**) *R*^2^ of single predictor linear models (light gray), and the final models incorporating all features (dark gray). *R*^2^ values were calculated on held out data in a 10-fold cross-validation strategy. Error bars are the SDs of the *R*^2^ values of the 10 folds. (**I**) Correlation between observed and predicted ∆∆*G*_a_ for the model incorporating all features.

We next considered the spatial organization of allostery by considering all individual mutations across the KD. The average effect of mutations on Src activity is much stronger closer to the Src active site (Fig. 4A and fig. S5A). Considering all 252 residues in the Src KD, there is an exponential decay of mutational effects on activity away from the active site (Fig. 4A) with a decay rate *k* = −0.063 ± 0.008 Å^−1^ (95% confidence interval), corresponding to a 50% reduction of allosteric effects over a distance *d*_1/2_ = 7.45 Å (Fig. 4A). The distance dependence is, on average, similar in the N and C lobes of the KD despite their structural differences (*k* = −0.074 ± 0.005 Å^−1^ for the N lobe and *k* = −0.075 ± 0.003 Å^−1^ for the C-lobe) (Fig. 4B). In contrast to what is observed for inactivating mutations (∆∆*G*_a_ > 0), whose effects scale with distance (*k* = −0.078 ± 0.004 Å^−1^) (Fig. 4D), activating mutations do not decay with distance (*k* = 0.011 ± 0.003 Å^−1^) (Fig. 4C). This effect is not driven by the smaller effect size of activating mutations as inactivating mutations with matched effect sizes have a stronger negative decay (median *k* = −0.028 Å^−1^, simulation *P* = 1 ×^−4^, *n* = 10,000 subsamples; fig. S5, C to E). This suggests different mechanisms underlie inhibitory and activating mutations.

However, allosteric communication in the KD is not isotropic as not all mutations occurring at a given distance have equal effects. Instead, mutations in particular residues and in particular directions from the active site have stronger effects on activity at a given distance. To illustrate this, the distance dependence when only considering the major allosteric sites is much weaker (*k* = −0.038 ± 0.005 Å^−1^, *d*_1/2_ = 18.24 Å) (Fig. 4A, inset, and fig. S5B). Major allosteric sites are not arranged uniformly throughout the KD. Instead, they are spatially clustered and have higher connectivity than expected by chance (fig. S5, F to I). Quantification of the allosteric decay rate from the active site in three orthogonal spatial axes [*x*, *y*, *z*, axes as defined in Protein Data Bank (PDB) ID: 2SRC] in the positive and negative directions (+, −) (Fig. 4D and fig. S5J), reveals more efficient transmission in the direction toward helix αC (*y*+, *k* = −0.035 ± 0.006 Å^−1^, *d*_1/2_ = 19.80 Å), and in the vertical axis of the KD (*z*+, *k* = −0.045 ± 0.004 Å^−1^, *d*_1/2_ = 15.40 Å and *z*−, *k* = −0.074 ± 0.007 Å^−1^, *d*_1/2_ = 9.37 Å), and less efficient transmission toward the regulatory domain interaction surfaces (*x*+, *k* = − 0.215 ± 0.026 Å^−1^, *d*_1/2_ = 3.22 Å). Allosteric transmission also differs across secondary structure types, with faster decay for mutations occurring in β strands, where mutations have smaller effects than expected given their distance to the active site (fig. S5, K and L).

Inhibitory allosteric communication in the Src KD is thus strongly distance dependent but also anisotropic: Transmission efficiency is dependent on the direction of propagation, with at least a sixfold difference in decay rates between the most and least efficient directions.

### Allosteric communication via dynamic noncovalent contacts

The conformation of the Src KD differs between its active and inactive states, with changes in the positioning of helix αC and the activation loop, and multiple residue contact rearrangements in the active site and throughout the KD (Fig. 4E) (*50*, *52*, *56*). On the basis of their contacts in active (PDB ID: 1Y57) and inactive (PDB ID: 2SRC) state structures of Src, we define four types of residues for different types of contacts (e.g., salt bridges, pi-cation interactions): active-only (engaging in contacts only in the active state), inactive-only (only in the inactive state), swapping (residues that have different contacts in the two states), and static (residues with the same contacts in both states) (Fig. 4E).

Overall, mutations in active-only and swapping residues are more likely to affect the activity of the Src KD than those in inactive state–only residues (Fig. 4F). The differences in |∆∆*G*_a_| are strongest when considering contacts between side chains, including salt bridges, pi-cation interactions, and side-chain to side-chain hydrogen bonds (Fig. 4F). Swapping residues include those forming Src’s “electrostatic switch network” of contacts that change during activation: D407-K298, E313-R412, and D389-Y419 in the inactive state, which break and rearrange into E313-K298 and R412-Y419 (Fig. 4F). Mutations in these residues are extremely detrimental for Src activity (Figs. 4F and 1K). The allosteric map thus shows that residues with contacts that change upon activation are particularly important for Src activation and enables the prioritization of which of these dynamic contacts are most important in the activation process.

### Amino acid changes and allostery

We next considered different types of mutation across the domain. Mutations at histidine, and several hydrophobic residues (alanine, isoleucine, valine, phenylalanine, and tryptophan) are more likely to have inhibitory effects (FDR < 0.1, FET) and substitutions to proline are the most likely to be inhibitory (FDR < 0.1, FET; Fig. 4G), as also observed for allosteric regulation of protein-protein interactions (*12*, *31*). In Src, substitutions to charged residues (aspartate, lysine, and arginine) are also more likely to be allosteric (Fig. 4G). Substitutions to cysteine, alanine, and methionine are the least likely to be allosteric (FDR < 0.1, FET; Fig. 4G). The smaller number of activating allosteric mutations makes analyses of their properties less powered. However, mutations at glutamate residues (FDR < 0.1, FET) and substitutions to hydrophobic amino acid are more likely to be allosteric activating mutations (OR = 1.61, *P* = 0.017, FET for hydrophobics as a group) (Fig. 4G).

### Predicting allostery

We next asked how much of the variance in allostery (∆∆*G*_a_) for mutations outside of the active site can be accounted for by sequence and structural features. We used linear modeling to predict ∆∆*G*_a_ from simple features: the minimum heavy atom distance of the mutated residue to the nucleotide [adenylyl-imidodiphosphate (AMP-PNP) in PDB structure 2SRC] and to the catalytic residue D389, the identity of the wild-type (WT) and mutant amino acid, solvent accessibility, contact type and dynamics (active-only, inactive-only, swapping, and static), and secondary structure element type. Distance to the catalytic site and to the nucleotide are the most predictive features when tested individually (Fig. 4H). A linear model combining all predictors explains 40% of the variance in ∆∆G_a_ (tested on held out data, 10-fold cross-validation, Fig. 4H), which increases further to 46% when incorporating specific secondary structure elements as a feature (tested on held out data, 10-fold cross-validation; Fig. 4, H and I). Mutation effects on activity are thus reasonably well-predicted from simple structural features alone, illustrating the potential to predict allostery from sequence.

### Prioritization of surface pockets

Most kinase inhibitors developed to-date target the highly conserved orthosteric ATP-binding site, resulting in limited specificity (*13*, *14*). Allosteric inhibitors have been successfully developed to increase specificity and overcome drug resistance mutations for several kinases including BCR-ABL (*21*), BRAF, MEK (*23*), and AKT (*24*). However, each kinase has many potentially druggable surface pockets and, beyond a small number of examples, it is not known which of these pockets are allosterically active in each kinase.

**Fig. 5.**
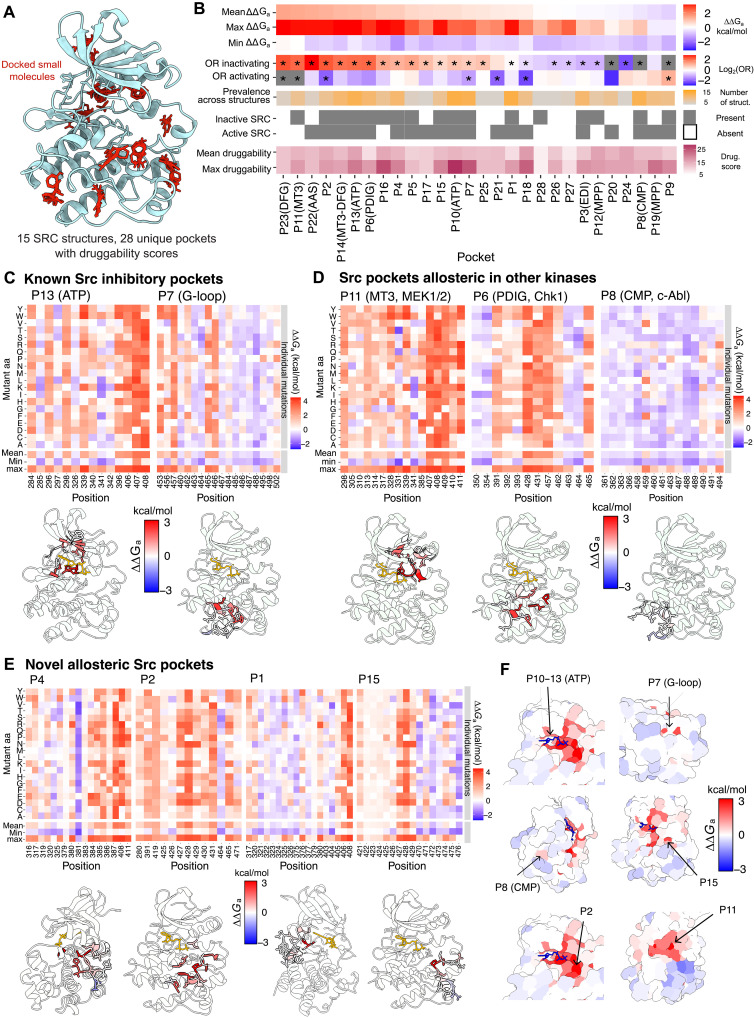
The druggable allosteric surface of Src. (**A**) Overview of the Kinase Atlas pocket dataset for the Src kinase, consisting of 15 Src structures with multiple small-molecule docking sites and their associated druggability scores. (**B**) Summary of the regulatory impact and druggability properties of Src surface pockets. Mean ∆∆*G*_a_ = average of per site–averaged ∆∆*G*_a_ of all residues in the pocket. Max ∆∆*G*_a_ = maximum of per site–averaged ∆∆*G*_a_ of all residues in the pocket. Min ∆∆*G*_a_ = minimum of per site–averaged ∆∆*G*_a_ of all residues in the pocket. Pockets significantly enriched or depleted in activating and inactivating mutations (FET FDR < 0.05) are labeled with stars. (**C** to **E**) Heatmaps showing ∆∆*G*_a_ of mutations in Src previously targeted pockets (C), in Src pockets homologous to pockets known to be allosteric and/or targeted by drugs in other kinases (D), and in previously unknown Src pockets (E). The residues forming each pocket are highlighted in the structure of Src (PDB ID: 2SRC), colored by their weighted mean ∆∆G_a_. Pockets previously targeted by drugs are labeled with a capsule pill. (**F**) Surface view of representative examples of Src pockets, including highly druggable and allosterically active pockets [P10–13(ATP), P7, P15, P2, P11], and highly druggable but allosterically inactive pockets [P8(CMP)].

Structural analysis of the surface of Src (see Materials and Methods) identifies 28 predicted small-molecule fragment-binding hotspots (henceforth referred to as surface pockets) present in at least 1 of 15 different Src structures (Fig. 5A and fig. S6) (*26*). To prioritize these pockets for drug development, we used our comprehensive atlas to quantitatively rank these 28 pockets based on their allosteric activity (Fig. 5B).

In total, 16 Src pockets are enriched for inhibitory mutations, with varying levels of enrichment (FET, FDR < 0.05; see Fig. 5B for a rank). These inhibitory pockets include the orthosteric ATP-binding site targeted by competitive inhibitors. Beyond the orthosteric site, two other surface pockets of Src have been targeted by small-molecule inhibitors: the DFG pocket (*57*) and P7 (*58*). Consistent with this, both of these pockets are enriched for allosteric inhibitory mutations in the allosteric map, genetically validating their regulatory potential (Fig. 5C).

### Previously unknown allosteric pockets

Across 538 protein kinases, 12 different pockets have been reported as potentially allosteric (*26*). Some of these pockets are the binding sites of small-molecule inhibitors, whereas others are physiological binding sites for other proteins and lipids or dimerization interfaces (*26*). Of these 12 pockets, 7 have a structurally homologous pocket in Src (table S8) (*26*), but it is unknown how many of these—if any—are also allosterically active in Src.

In total, four of these seven pockets are enriched for inhibitory allosteric mutations (FDR < 0.05, FET; Fig. 5, B and D, and fig. S8). Pockets that are allosteric in other kinases and also strongly allosteric in Src include P11, homologous to the MT3 pocket in MEK1/2 targeted by type III allosteric inhibitors (*23*); P22, homologous to the AAS site in Aurora A, where one KD activates another through binding of its activation segment to this site (*59*); and P6, homologous to the PDIG pocket in CHK1 close to the substrate binding site that is bound by small molecule inhibitors (Fig. 5, B and D) (*60*). In contrast, three other pockets homologous to allosteric pockets in other kinases show little evidence of allostery in Src. These include pocket P3 (Fig. 5B), homologous to the EDI site that is part of the EGFR dimerization interface and P8 (Fig. 5, B and D), homologous to the Bcr-Abl myristoyl pocket (CMP) that has been targeted by small-molecule inhibitors (*21*). Three of the residues that form the myristoyl pocket in Abl are substituted by bulky residues in Src (*61*), likely rendering the pocket unable to bind myristate (*61*) and consistent with the pocket having little evidence of allosteric activity in our dataset.

Last, we identified multiple surface pockets of Src that, to our knowledge, have not been previously reported as allosteric in any kinases (*26*). A total of 10 additional pockets are enriched for allosteric mutations in Src (Fig. 5, B and E, and fig. S8). Nine of these previously unknown pockets are enriched for inhibitory allosteric mutations and one (P9) is enriched for activating allosteric mutations. These novel allosteric pockets include P4, P16, and P25 located between the allosteric αC helix and the active site; P1 formed by residues in the αC-β4 loop, αE and β8, and P18 and P21 in the surface of the N-lobe β sheet; and P2, P15, and P5 located on both sides of αEF and the substrate positioning loop (see Fig. 5E, for examples, and fig. S10 for a visualization of pocket overlaps at a range of clustering thresholds). Computational methods (*62*–*65*) do not successfully predict the allosteric pockets in Src (fig. S9). A full list of all pockets with druggability and inhibitory and activatory allosteric scores is available in table S3, and full surface views of the KD depicting per-residue weighted mean and maximum weighted ∆∆*G*_a_ are available as movies S4 and S5, respectively.

The comprehensive mutational effect data therefore serves to genetically prioritize which of the many potentially druggable surface pockets of Src should be the focus for inhibitory and activatory drug discovery. Moreover, the allosterically active pockets in Src identify multiple previously unknown pockets not previously demonstrated as allosteric in any kinases (fig. 5E), suggesting hitherto unappreciated opportunities for developing allosteric kinase modulators.

### Modulation of the allosteric landscape by the Src regulatory domains

**Fig. 6.**
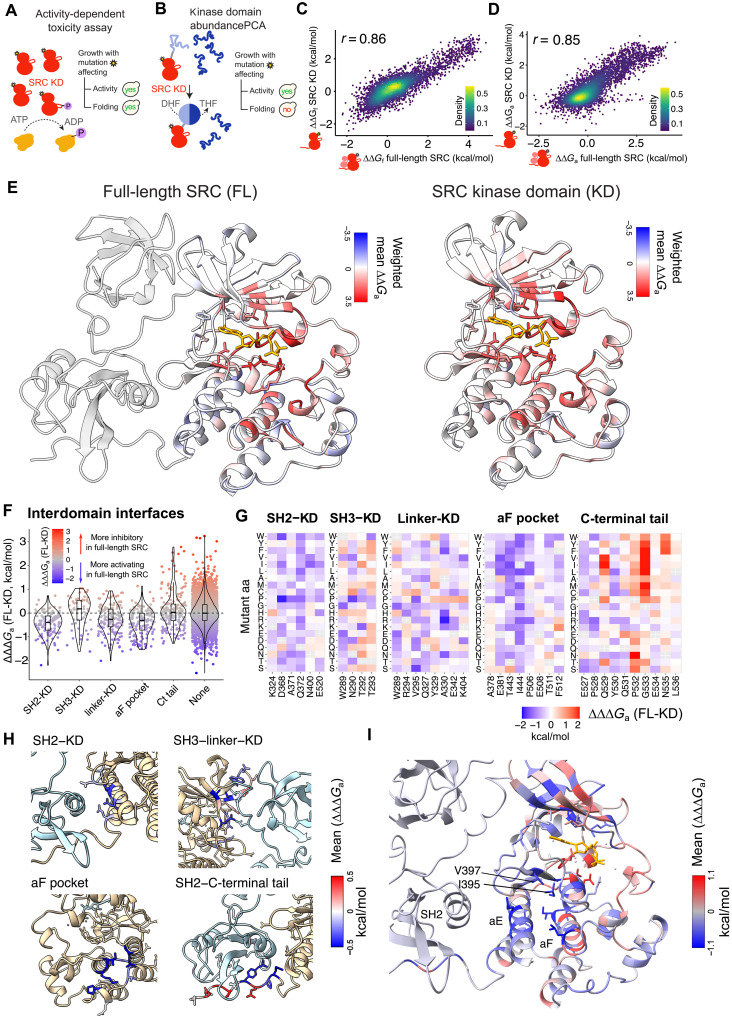
Impact of Src regulatory domains on the allosteric landscape. (**A**) Overview of the toxicity selection assay to measure the protein kinase activity of Src KD variants at scale. (**B**) Overview of the abundancePCA (aPCA) selection assay to measure in vivo abundance of Src KD variants at scale. (**C**) Correlation between inferred ∆∆*G*_f_ in full-length Src and ∆∆*G*_f_ in Src KD. (**D**) Correlation between inferred ∆∆*G*_a_ in full-length Src and ∆∆*G*_a_ in Src KD. (**E**) Structural view of the allosteric landscape of the Src KD in the context of full-length SRC (left structure) and in isolation (right). (**F**) Distribution of changes in ∆∆G_a_ between full-length and KD Src (∆∆∆*G*_a_) at regulatory domain interfaces. (**G**) Heatmaps showing changes in ∆∆G_a_ between full-length and KD Src (∆∆∆*G*_a_) at regulatory domain interfaces with the KD. (**H**) Src structures (PDB ID: 2SRC) with residues in regulatory domain interfaces colored by mean ∆∆∆*G*_a_. (**I**) Src structure colored by mean ∆∆∆*G*, with the secondary structure elements and containing the C-lobe cluster of residues more activating in full-length Src labeled.

Src, like most kinases and eukaryotic proteins, is a multidomain protein. In addition to the catalytic KD, Src contains two additional globular domains, Src homology 1 domains 2 (SH2) and SH3, disordered linkers, and the dynamic SH4 region. The noncatalytic domains of Src physically interact with the KD in its inactive conformation and inhibit activity (*50*, *56*, *66*). To ask how the regulatory domains of Src affect allosteric communication in the catalytic domain, we repeated our abundance and activity selections for the same 54,455 Src variants in the KD in the absence of Src regulatory domains (Fig. 6, A and B). The selections were highly reproducible (median *r* = 0.90 for activity and *r* = 0.75 for abundance; fig. S7A), and the fitness scores correlated well with independent in vivo activity (*r* = 0.89) and abundance (*r* = 0.66) measurements (fig. S7, B and C). Inferred ∆∆*G*_f_ correlated well with *K*_50_ values derived from in vitro protease sensitivity experiments (Pearson’s *r* = −0.84, *P* = 1.4 ×^−10^, *n* = 27 variants; fig. S7D).

Fitting the same three-state folding and activation model to the Src KD data allows us to quantitatively compare the effects of all 5111 single amino acid substitutions on stability and activation in the presence and absence of the Src regulatory domains (fig. S7, E to H, and movies S6 to S10). Overall, the mutational effects on folding energies and activity energies correlate very well between the KD and full-length Src constructs (*r* = 0.86 and *r* = 0.85, respectively; Fig. 6, C to E). The allosteric landscape of the KD is therefore highly conserved in the presence of the regulatory domains (Fig. 6E). Accordingly, the enrichment of inhibitory mutations in surface pockets is largely conserved in the absence of the regulatory domains resulting in nearly identical pocket prioritization for Src inhibition (fig. S8).

However, there are differences between the two allosteric landscapes. Activating mutations are more frequent in full-length Src than in the KD alone (Fig. 6E and fig. S7I), consistent with the regulatory domains having an overall inhibitory function (*50*, *56*, *66*). In particular, mutations in the interdomain surfaces with the SH2 domain and the SH2-KD linker have stronger activating effects in the full-length kinase (Fig. 6, F to H), consistent with these intramolecular interactions inhibiting kinase activity. Mutations in the αF helix pocket, proposed to bind the SH4 region for additional inhibition of Src activity (*44*), also more strongly activate full-length Src. These differences are not driven by changes in the effects of mutations on abundance between full-length Src and the KD alone, as fitting the three-state folding and activation models exchanging the underlying abundance data does not affect the conclusions (fig. S7J).

Mutations in the dynamic C-terminal tail of Src also differ in their effects between full-length Src and the KD alone (Fig. 6, F to H). Mutations in Y530, the inhibitory phosphosite directly involved in the interaction with the SH2 domain, have stronger activating effects in full-length Src (∆∆∆*G*_a_ < −1, FDR < 0.1; see Materials and Methods), consistent with a release of the inhibitory interaction. The adjacent E527, P528, and Q531 are similarly enriched for mutations with stronger activating effects in full-length Src. In contrast, Q529, P532, G533, and N535 are enriched for mutations with stronger inhibitory effects in full-length Src (∆∆∆*G*_a_ > 1, FDR < 0.1) (Fig. 6, F to H). Many inhibitory mutations match the in vitro phosphorylation motif of Src (*67*), including Y, L, and V at position −1 (Q529), and Y, F, W, I, and L at position +3 (G533). This suggests that in the absence of Csk-mediated phosphorylation in yeast, autophosphorylation of Src at the C-terminal tail can elicit autoinhibition, consistent with recent reports of intermolecular autophosphorylation of Src Y530 (*68*). We find additional inhibitory mutants not matching Src in vitro motif preferences, including mutation to N at +2, and mutation to Y, F, and W at +5. This may reflect strong context dependence of Src motifs not captured in peptide arrays; alternatively, these variants may act by increasing the affinity of the tail for the SH2 domain of Src (*50*, *56*, *66*) independently of phosphorylation.

Last, spatial clustering of mutations with stronger activating effects in full-length Src (∆∆∆*G*_a_ < −1, FDR < 0.1) reveals an additional cluster of residues in the C lobe (fig. S7K, and Fig. 6I). This cluster includes the SH2-KD interface on the outer surface of αE, along with the internal surface of αE, a second internal layer of residues in αF pointing toward αE, and the C-spine residues I395 and V397 (Fig. 6E). Mutations in these sites at a distance from the SH2 domain interface are thus more activating in full-length Src, potentially allosterically relieving inhibition by the SH2 domain.

## DISCUSSION

We have presented here a comprehensive map of positive and negative allosteric regulation of an enzyme. The Src allosteric atlas provides a complete map of perturbations that inhibit and activate enzymatic activity and a framework to guide the future development of therapeutics. The map identifies multiple accessible surface allosteric sites that have not, to our knowledge, been previously reported in any kinases. These sites are also not predicted by existing computational methods (fig. S9) (*62*–*65*), demonstrating that experimental mapping can identify many previously unknown accessible allosteric sites even in highly studied proteins. The abundance of novel allosteric sites in Src suggests that the target space for the development of allosteric modulators is large and still largely unexplored. We believe that this is likely to be the case for many protein families.

Allosteric communication occurs throughout the KD of Src but it is not equally efficient in all directions. The exponential decay of allostery with three dimensional distance is consistent with measurements of energetic couplings and structural perturbations (*69*) and may explain evolutionary conservation gradients away from enzyme active sites (*70*). The enrichment of allosteric mutations in residues with active state and dynamic structural contacts suggests that at least some of the anisotropy in allostery is due to shifts in conformational ensembles (*8*).

Like many kinases, the full-length Src protein contains multiple regulatory domains. Recharting the complete allosteric map in the presence of these domains revealed that allostery is strikingly conserved, with important shifts at only a subset of sites, in particular regions contacting and close to the regulatory domains.

The approach that we have taken here extends allosteric mapping (*12*, *31*) to many classes of proteins important for medicine and biotechnology. The main requirement is for a selection assay that links genotype to enzymatic activity. Microfluidic (*34*), complementation (*36*), and toxicity assays (*35*) exist for many enzymes, and the specific selection used here can be applied to many kinases encoded by human disease genes (*45*, *71*).

An interesting direction for future work will be to generate allosteric maps in homologous proteins. This will allow fundamental questions about the conservation and evolution of allosteric sites to be addressed. It will also allow the identification of allosteric pockets functionally or structurally conserved in only one or a subset of proteins in a family, potentially allowing the development of more specific therapeutics.

We believe that mapping allostery in enzymes will prove to be an efficient experimental strategy to elucidate the mechanistic principles of allostery and to generate datasets of sufficient size and diversity to train machine learning models to predict allosteric sites. We have shown here that simple structural and sequence features perform reasonably well for predicting allosteric mutations in Src. By quantifying allosteric communication in structurally diverse proteins, we should be able to train computational models to predict, therapeutically target, and engineer allosteric sites in any protein.

## MATERIALS AND METHODS

### Media

The media used in the study are as follows:

1) LB: Bacto-tryptone (10 g/liter), yeast extract (5 g/liter), and NaCl (10 g/liter). Autoclaved for 20 min at 120°C.

2) YPDA: glucose (20 g/liter), peptone (20 g/liter), yeast extract (10 g/liter), and adenine sulphate (40 mg/liter). Autoclaved for 20 min at 120°C.

3) SORB: 1 M sorbitol, 100 mM LiOAc, 10 mM tris (pH 8.0), and 1 mM EDTA.

4) Filter sterilized (0.2 mm nylon membrane, Thermo Fisher Scientific).

5) Plate mixture: 40% PEG3350, 100 mM LiOAc, 10 mM tris-HCl (pH 8.0), and 1 mM EDTA (pH 8.0). Filter sterilized.

6) Recovery medium: YPD [glucose (20 g/liter), peptone (20 g/liter), and yeast extract (10 g/liter)] +0.5 M sorbitol. Filter sterilized.

7) SC-URA: yeast nitrogen base (6.7 g/liter) without amino acid, glucose (20 g/liter), and complete supplement mixture drop-out without uracil (0.77 g/liter). Filter sterilized.

8) SC-URA/ADE: yeast nitrogen base without amino acid (6.7 g/liter), glucose (20 g/liter), complete supplement mixture drop-out without uracil (0.76 g/liter), adenine, and methionine. Filter sterilized.

9) MTX competition medium: SD-URA/ADE + methotrexate (200 μg/ml) (BioShop Canada Inc., Canada), and 2% dimethyl sulfoxide (DMSO).

10) SC-URA 2% raffinose 0.1% glucose: yeast nitrogen base without amino acid (6.7 g/liter), raffinose (20 g/liter), glucose (1 g/liter), and complete supplement mixture drop-out without uracil (0.77 g/liter). Filter sterilized.

11) SC-URA 2% galactose 0.1% glucose: yeast nitrogen base without amino acid (6.7 g/liter), galactose (20 g/liter), glucose (1 g/liter), and complete supplement mixture drop-out without uracil (0.77 g/liter). Filter sterilized.

12) DNA extraction buffer: 2% Triton X-100, 1% SDS, 100 mM NaCl, 10 mM tris-HCl (pH 8), and 1 mM EDTA (pH 8).

### Plasmid construction

All the Src constructs for expression in yeast and plasmid sequences have been verified by whole-plasmid sequencing (Plasmidsaurus). Their sequences and Benchling links can be found in table S4. All oligonucleotide sequences used for plasmid construction can be found in table S5. Full-length and KD Src sequences used can be found in table S6.

To assay in vivo soluble expression of the Src KD, we used pGJJ133 (selection markers: Amp, URA), a modified version of the pGJJ045 aPCA plasmid where stop codons have been introduced downstream of the NheI-HindIII cloning sites to allow flexible cloning of open reading frames that do not contain STOP codons. To assay activity-dependent toxicity of Src, we used pTB022 (Selection markers: Amp, URA), a plasmid based on the same backbone as the aPCA plasmids but containing a yeast GAL promoter to drive the expression of NheI-HindIII inserts not fused to any DHFR fragment or linker. We ordered a gene block of Src codon-optimized for yeast expression and flanked by NheI-HindIII restriction sites (IDT). We used oligonucleotides oTB063 and oTB064 to amplify the Src KD and introduce an NheI site at the 5′end of the KD sequence, and we cloned the KD fragment (positions 261 to 536) on pGJJ133 via restriction digestion and T4 ligation with NheI and HindIII (NEB), resulting in pTB109. The KD fragment was cloned on pTB022 via restriction digestion with NheI and HindIII (NEB) and T4 ligation (NEB), and the resulting plasmid was subjected to a round of site-directed mutagenesis (NEB) with oTB215 and oTB216 to introduce a start codon at the beginning of the KD sequence, resulting in pTB112.

To assay in vivo soluble expression of full-length Src, we generated pTB043 (selection markers: Amp, URA) via Gibson assembly. pTB043 is based on the same backbone as the aPCA plasmids and contains a construct where full-length Src is fused to the DHFR3 fragment in its N terminus and to the DHFR1,2 fragment in its C terminus. This fusion construct is driven by a cyc promoter and terminated by a cyc terminator, as in the rest of aPCA plasmids. To assay activity-dependent toxicity of full-length Src, we cloned the Src gene block into pTB022 using NheI-HindIII restriction digestion and T4 ligation, resulting in pTB023.

### Variant library design and cloning

To fully cover the Src KD from E268 to L536, the library was divided in five overlapping blocks or tiles of ~60 amino acids to be cloned and selected separately. Within each tile, we chose 10 genetic backgrounds with a wide range of effects on Src kinase activity based on a previous deep mutagenesis dataset (*44*). The sequences of all tiles, constant regions, and genetic backgrounds are in table S4.

The library was ordered as two IDT oPools (pool 1 with block1 and pool 2 with blocks 2 to 5), containing all NNK single mutants in each of the 10 backgrounds of each block. oPool material (2.5 μl 0.1 μM) was used as a template in a 100 μl of Q5 high-fidelity 10 cycle polymerase chain reaction (PCR) reactions. Primers specific to the constant regions of each block were used (see oligonucleotides). The amplified products were verified on an agarose gel and column-purified (QIAquick PCR purification kit, QIAGEN) for Gibson assembly. The library was assembled on pTB112 (KD) and on pTB023 (full-length). To do so, the plasmids were linearized with primers pointing outward from the constant regions of each block (bb primers, see oligonucleotides) so that each linearized vector had at least 20 nt of homology to the amplified oligo pool containing the variants. One hundred and twenty nanograms of linearized plasmid was assembled with the amplified oligo pools in a 20 μl Gibson reaction using in-house prepared Gibson assembly mix, and incubating for 3 hours at 50°C. The reaction products were dialyzed using 0.025 μM mixed cellulose ester (MCE) membranes, concentrated to 5 μl using a SpeedVac machine, and transformed into NEB 10β High-efficiency Electrocompetent *Escherichia coli* cells according to the manufacturer’s protocol. Cells were left to recover in SOC medium (NEB 10β Stable Outgrowth Medium) for 30 min, a 2-μl aliquot was plated to quantify the total number of clones, and the rest of the cell volume was transferred to 100 ml of LB medium with ampicillin overnight. One hundred milliliters of each saturated *E. coli* culture were harvested the next morning to extract the plasmid library using the Plasmid Plus Midi Kit (QIAGEN). The assemblies were verified using Sanger sequencing (Eurofins). The assembled mutated Src library constructs were transferred into the pGJJ133 and pTB198 plasmids using NheI-HindIII digestion (NEB) and overnight temperature cycling T4 ligation (NEB), followed by dialysis, electroporation, overnight LB-ampicillin selection, and plasmid isolation as described above.

### Large-scale transformation and competition assays

Each library corresponding to each of the five blocks was transformed in triplicate, and with a coverage of ~100× or greater. Three 500-ml YPDA cultures of late log phase *Saccharomyces cerevisiae* BY4741 cells [optical density (OD) ~ 0.8 to 1] were harvested in 50-ml Falcon tubes, each resuspended in 22-ml SORB medium, and incubated for 30 min on a shaker at room temperature (RT). Single-stranded DNA [437.5 μl, 10 mg/ml previously boiled (5 min, 100°C)] was added to the cells, and the mix was separated in 5 aliquots of 4.3 ml in 50 ml Falcon tubes, one for each library block. Three micrograms of library plasmid DNA was added to each aliquot, followed by 17.5 ml of plate mixture, and the mix was incubated for 30 min on a shaker at RT. DMSO (1.75 ml) was then added, and the cells were incubated at 42°C for 20 min. Following incubation, the cells were centrifuged for 3 min at 3000*g*, the supernatant was discarded with a pump, and the cells were resuspended in recovery media and incubated at 30°C for 1 hour. The cells were then centrifuged for 3 min at 3000*g* and transferred into 100 ml of SC-URA. Ten microliters of of this culture was immediately plated onto SC-URA selective plates to monitor transformation efficiency. The rest of the culture was incubated overnight at 30°C.

For the activity-dependent toxicity assays, the overnight SC-URA cultures were used to inoculate the next day a 100-ml culture of SC-URA with 2% raffinose and 0.1% glucose at an OD = 0.2 to 0.4, which was grown overnight. Cells from this culture were inoculated the next day in 100 ml of SC-URA with 2% galactose and 0.1% glucose at an OD = 0.05, to induce overexpression of the Src variant library. The remaining input cells grown in 2% raffinose and 0.1% glucose were harvested and frozen for DNA extraction (inputs). The galactose cultures were left to grow overnight to an OD = 1.6 to 2.5, corresponding approximately to five generations, harvested, and frozen for DNA extraction (outputs).

For the aPCA and sPCA assays, the overnight SC-URA cultures were used to inoculate the next day a 100-ml culture of SC-URA-ADE at an OD = 0.2 to 0.4, which was grown overnight (input culture). Cells from this culture were inoculated in 100 ml of SC-URA/ADE + MTX (200 μg/ml) to select stably expressed Src variants. The remaining input cells grown SC-URA/ADE were harvested and frozen for DNA extraction. The MTX cultures were left to grow overnight to an OD = 1.6 to 2.5, corresponding approximately to five generations, harvested, and frozen for DNA extraction (outputs).

### DNA extraction, plasmid quantification, and sequencing library preparation

Total DNA was extracted from yeast pellets equivalent to 50 ml of cells at OD = 1.6 as described in our previous work (*12*, *31*). Plasmid concentrations in the resulting samples were quantified by against a standard curve of known concentrations by quantitative PCR (qPCR), using oGJJ152 and oGJJ153 as qPCR primers that amplify in the origin of replication of both toxicity and aPCA assay plasmids.

To generate the sequencing libraries, we performed two rounds of PCR amplification. In the first round, we used primers flanking the mutated regions in each sample (five pairs of PCR1 primers, one for each of the Src blocks). This PCR1 reaction allows increasing the nucleotide complexity of the first sequenced bases by introducing frame-shift bases between the Illumina adapters and the sequencing region of interest. For block 5, a different reverse frameshifting pool was used for the sPCA libraries as they differ in the region downstream of the STOP codon of Src (oTB470^+^ was used instead of oGJJ589^+^). All frameshifting PCR1 primers are listed in the Oligonucleotides table. A total of 125 million plasmid molecules were used as templates and were amplified for eight cycles. The reactions were column-purified (QIAquick PCR purification kit, QIAGEN), and 40 ng of DNA was used as template for a PCR2 reaction with the standard i5 and i7 primers to add the remainder of the Illumina adapter sequences and the demultiplexing indices (dual indexing) unique to each sample. This PCR2 was run for eight cycles, and the resulting amplicons were run on a 2% agarose gel to quantify and pool the samples for joint purification, and to ensure the specificity of the amplification and check for any potential excess amplification problems. The final libraries were size selected by electrophoresis on a 2% agarose gel, and gel-purified (QIAEX II Gel Extraction Kit, QIAGEN). The amplicons were subjected to Illumina paired-end 2 × 150 sequencing on a NextSeq2000 instrument.

### Sequencing data processing and thermodynamic modeling

FastQ files from paired-end sequencing of all aPCA and toxicity experiments were processed with DiMSum (*72*) v1.2.11 (https://github.com/lehner-lab/DiMSum) using default settings with minor adjustments. A minimum input read count threshold was set for 1-nt substitutions using the “fitnessMinInputCountAny” option to minimize the fraction of reads per variant related to sequencing error-induced “variant flow” from the WT. The option “barcodeIdentityPath” was used to specify a variants file to recover only the variants corresponding to the designed library (NNK mutations in one of the predefined genetic backgrounds).

We used MoCHI (*32*) (https://github.com/lehner-lab/MoCHI) to fit two global mechanistic models, one for the Src KD and one for full-length Src, using the corresponding 10 sPCA/aPCA and toxicity assay datasets (2 molecular phenotypes × 5 blocks) simultaneously. The software is based on our previously described genotype-phenotype modeling approach (*12*) with additional functionality and improvements for ease-of-use and flexibility (*31*, *32*).

We fit a phenomenological enzyme folding and activation model to the Src kinase data. To do so, we use an explicit three-state thermodynamic model in which the protein can exist in three states: unfolded and inactive (ui), folded and inactive (fi), and folded and active (fa). The folding energy, ∆*G*f, is defined as the energy difference between the unfolded (ui) and folded (fi) inactive states, and the “activity” energy, ∆*G*_a_, is defined as the energy difference between the inactive (fi) and active (fa) folded states. We note that this ∆*G*_a_ parameter quantifies all changes in the activity of Src not explained by changes in abundance, including changes in the equilibrium between active and inactive conformations of the enzyme, but also any other effects on catalysis (*k*_cat_) or substrate specificity (*k*_m_) not related to conformational changes. We assume that the probability of the unfolded and active state (ua) is negligible and that changes in folding and active state energies are additive, i.e., the total free energy change of an arbitrary variant relative to the WT sequence is simply the sum over residue-specific energy changes corresponding to all constituent single amino acid substitutions.

We set MoCHI parameters to specify a neural network architecture consisting of two additive trait layers, one for folding and one for active state energies, as well as one linear transformation layer per experiment (5× for toxicity and 5× for aPCA blocks). The specified nonlinear transformations “TwoStateFractionFolded” and “ThreeStateFractionBound” derived from the Boltzmann distribution function relate energies to proportions of folded molecules, and molecules in the active versus inactive states of Src respectively. We used as input data the WT, single, and double amino acid variants of Src (--order_subset 0, 1, 2) and modeled the data in the absence of interactions between single substitutions (--max_interaction_order 1). DiMSum output fitness tables were formatted to include the full five-block sequence, and the sign of the toxicity assay fitness was reversed as Src activity and cellular fitness are anticorrelated. We additionally removed variants corresponding to the activity dead and unstable L494P genetic background as the fitness range of this background is extremely narrow, and we find it greatly biased block five fitted energies toward negative values. The training and validation workflow, as well as the confidence estimation used was as described in our previous work (*12*, *31*) but without L2 regularization as we did not observe artifactually large values of fitted free energy changes. For model performance estimates, the maximum explainable variance (MEV) (of genetic origin) was calculated by subtracting the total estimated technical variance (as reported by DiMSum fitness errors) from the total fitness variance, i.e., $\mathrm{MEV}=\mathrm{Var}\left(\text{fitness}\right)-\frac{1}{n}\sum_{i}^{n}\sigma_{i}^{2}$, where $\sigma_{i}$ is the fitness error associated with variant i and n is the total number of variants. The fraction of explainable variance (FEV) is then given by FEV=MEV/Var(fitness). To minimize the effect of outlier fitness errors on FEV estimates, we discarded variants whose errors were in the top 15th percentile. We evaluated performance on the retained variants and computed the explainable variance captured by MoCHI models as the predicted versus observed *R*^2^ on held out double mutants divided by the FEV. We additionally fit a two-state model following the same procedure as above but where mutant effects on both activity and abundance fitness are assumed to be driven by a single underlying biophysical trait (TwoStateFractionFolded), and a four-state model in which mutations can have independent effects on two distinct active state conformations (“FourStateFractionBound”).

### Validation of ∆∆*G*_f_ using an mRNA-display protease sensitivity assay

We measured KD fold stability with an orthogonal high-throughput assay based on mRNA display and resistance to proteolysis in vitro (*39*). We used a human Src KD construct lacking the C-terminal tail (positions 265 to 526, see table S9 for construct and oligonucleotide sequences), in which we cloned the block 3 library.

The DNA library was in vitro transcribed using the T7 MEGAscript transcription kit. Four microliters of TURBO deoxyribonuclease (2 U/μl) was added, and the reaction was incubated for 15 min at 37°C. mRNA was purified using the NEB Monarch Spin RNA Cleanup kit. One nanomole of mRNA was then ligated with 10 μl 100 μM puromycin linker and 10 μl 100 μM splint oligo in a 100 μl reaction using 10 μl T4 ligase. The ligation products were next purified using ultrafiltration Amicon 50 kDa columns (UFC505096) by performing two centrifugation steps with 400-μl urea (7 M) and two further washes with nuclease-free water. The pure ligation product was then used in a PURExpress (E6800L) 175-μl translation reaction with 420-pmol mRNA used as input. To remove bound ribosomes to the mRNA-protein complexes, 20 μl of 30 mM EDTA was added, and the mixture was kept at RT for 20 min. Next, 20 μl 300 mM MgCl_2_ was added to neutralize EDTA. To perform reverse transcription Superscript IV, Thermo Fisher Scientific kit (18090050) was used. The reaction mix was kept at 45°C for 30 min. To remove EDTA and translation machinery, the protein-cDNA complexes were buffer exchanged with phosphate-buffered saline (PBS, pH 7.5) using ultrafiltration Amicon 50 kDa columns (UFC505096). The eluate was added to His Mag Sepharose Ni (Cytivia, GE28-9673-90) and incubated at 4°C for 30 min on a rotor. Protein-cDNA complexes were then eluted using 20 mM imidazole buffer and then buffer exchanged to PBS using ultrafiltration Amicon 50 kDa columns (UFC505096). The purified proteins were diluted in PBS, aliquoted, and snap frozen before storing at −80°C.

The proteolysis-based stability measurements were performed using Trypsin-EDTA (0.25%) with phenol red (Thermo Fisher Scientific). A maximum concentration of 43.3 mM trypsin was used to prepare 11 reactions in threefold dilution steps and one reaction with no protease. Twenty microliters of protein-cDNA complexes was added to each reaction. The mixture was kept at RT for 5 min before the reaction was stopped with 200 μl of 2% chilled bovine serum albumin solution in PBS. Next protein-cDNA complexes from each protease condition were purified using His Mag Sepharose Ni (Cytivia, GE28-9673-90).

cDNA concentration in each eluate was quantified by qPCR using PowerUp SYBR Green Master Mix (Thermo Fisher Scientific). A two-step PCR reaction as described for aPCA experiments was used to amplify the block 3 region (see oligonucleotides in table S5). The 12 PCR products were quantified using Agilent Tapestation and pooled in equimolar amounts for sequencing on an Illumina Novaseq 6000 platform.

Sequencing data were processed using DiMSum as described above for aPCA experiments. Sequencing counts at each trypsin concentration were used to quantify log_10_(*K*_50_) values using the Bayesian inference pipeline and scripts described in (*39*) (https://github.com/Rocklin-Lab/cdna-display-proteolysis-pipeline). The inferred log_10_(*K*_50_) values, which define the concentration of protease required for half maximal cleavage for each variant, were compared with our aPCA-inferred ∆∆*G_f_* using Pearson’s *r*, for variants with at least 10,000 counts in the no protease mRNA display samples (*n* = 27 variants).

### Identification of activating, inactivating, stabilizing and destabilizing mutations, and enrichments in secondary structure elements or functional regions

The mean weights (“mean kcal/mol”) and SDs (“SD kcal/mol”) from MoCHI fits were used for statistical testing to identify mutations with changes in stability or activity, using *z* tests, where Z=(ref.value−mean)/SD. *P* values were calculated on the basis of a normal distribution. Enrichments of particular mutation classes in individual sites or in subgroups of residues based on structural or functional annotations were tested using FET and comparing to the rest of the KD as background unless specified otherwise. In all box plots shown throughout the manuscript, the central line represents the median, the lower and upper hinges correspond to the first and third quartiles, the upper whisker extends from the hinge to the largest value no further than 1.5 * IQR (interquartile range) from the hinge, and the lower whisker extends from the hinge to the smallest value at most 1.5 * IQR of the hinge.

### Quantification of the distance dependence of mutation effects

To quantify the dependence of mutation effects on the distance from the active site, we computed the minimum distance between all atoms of each residue of Src and the active site using the 2SRC (*66*) structure. We computed distances to (i) the nucleotide (the nonhydrolyzable ATP analog AMP-PNP in 2SRC), (ii) the catalytic D388, and (iii) the proposed phosphosite substrate positioning residue P428 (*50*), and took the minimum distance for each residue to these three reference points. To fit an exponential curve to the data, we used the R stats package. We first used the optim() function to select reasonable starting values to then fit the $y=a\cdot e^{\mathrm{bx}}$ curve using the nls() function, where *a* is the estimate of |∆∆*G*_a_| at the active site (|∆∆*G*_a_|_0_), *b* is the decay rate (*k*), and *x* is the minimum heavy atom residue distance to the active site. To estimate distance-corrected mutation effects, we used the msir package (*73*) to fit a loess smoothing curve and quantified the residuals to the fit across different secondary structure element types. To quantify allosteric decay rate variation across different spatial directions, we used the *x*, *y*, and *z* directions as defined in the 2SRC pdb entry. To quantify decay in each direction, we considered residues at a distance of 10 Å or less from the active site in the two remaining orthogonal directions.

To compare the distance dependence of activating and inactivating mutations independently of their effect sizes, we subsampled the set of mutations with positive ∆∆*G*_a_ to match the number and distribution of effects of those with negative ∆∆*G*_a_ (*n* = 10,000 subsamples, with replacement), and fit exponential decays to these. A *P* value was calculated as the fraction of subsamples in which the decay is lower or equal than that of activating mutations.

To test for clustering of allosteric sites, we computed the distribution of C_α_-C_α_ pairwise distances between all allosteric sites in the Src KD and compared it to a null distribution obtained from 1000 subsets of randomly sampled residues of equal number. A *P* value was calculated as the fraction of subsets in which the median distance is lower or equal to the observed median distance. To test for connectivity, we took a similar approach but computing the distribution of minimum distances from each allosteric site to any other allosteric site.

### Quantification of structural features and contacts

The locations of individual secondary structure elements and functional annotations were obtained from (*50*, *74*). Solvent accessible surface area were calculated using freeSASA v2.0.3 (*75*) with parameters -n 20 --format = rsa --radii = naccess, and with 2SRC as a reference structure, both using the full-length Src structure, and using the KD residues alone. Secondary structure type annotations (helix, β sheet, turn, and loop) from UniProt were used.

To define active and inactive state contacts, we used getcontacts (https://getcontacts.github.io/) on representative structures of the inactive (2SRC) and active [1Y57 (*56*)] states. Before defining contacts, we added hydrogen atoms to the structures using the pymol h_add method. We then used get_static_contacts.py with parameters --itypes all. For the following analyses, we considered only salt bridges, pi-cation interactions, side chain–side chain hydrogen bonds, and side chain–backbone hydrogen bonds as the rest of contact types did not display conformational state specificity. Contacts of the same type and between the same residues were collapsed into a single contact, and duplicated contacts annotated both as salt bridge and side chain–side chain hydrogen bond were collapsed as salt bridge. We defined four types of residues based on their contact patterns: active residues engaged in contacts exclusively in the active state, inactive residues engaged in contacts only in the inactive state, nonspecific residues engaged in the same contact in both structures, and swapping residues that engage in different contacts in the active and inactive conformations. To display the contacts in Src structures, we used a pseudobond representation in ChimeraX (*76*), with the thickness of the contact being proportional to the averaged mutation effect of the two contacting residues. Contacts between swapping residues were represented as dashed pseudobonds.

### Allostery prediction

We fit linear models to predict ∆∆G_a_ using the base R lm() function, using as predictors the wt and mutant amino acid, secondary structure type in which the mutation is located, the specific secondary structure element in which the mutation is located, rSASA, contact type, and the residue type classification (active, inactive, swapping, both, or none) according to their contact patterns as described above. We also used as predictors the distance to D389 (catalytic site), and to the nucleotide (AMP-PNP in 2SRC), transformed according to the fitted exponential decay: $d_{\mathrm{tr}}=e^{k*d}$ where *d*_tr_ is the transformed distance, *k* = −0.063 Å^−1^, and *d* is the distance.

Models were evaluated on held out data using a 10-fold cross validation strategy. To evaluate the performance of allosteric site prediction models on predicting the Src dataset, we generated allosteric pocket predictions with allositePro (*63*), PASSer (*64*), and APOP (*65*), and compared the pocket scores of these predictors to the mean averaged ∆∆*G*_a_ of all constituent residues. In addition, we generated residue-level allosteric coupling intensity predictions (ACI) with Ohm (*62*) and compared them directly to ∆∆*G*_a_.

### Analysis of Src surface pockets

We used the Kinase Atlas (*26*) to retrieve all possible Src potentially druggable surface pockets. We used the docking analyses of all 15 available Src structures, and we defined each potential Src surface pocket as the set of residues located at a minimum distance of 5 Å from a cluster of docked molecules, resulting in a total of 384 pockets distributed across the 15 structures. After filtering out pockets with a druggability score < 5, we collapsed the remaining 254 pockets into a final set of unique pockets as many are present in multiple structures. To do so, we calculated a pairwise distance matrix between all pockets, using as a distance metric 1 minus the Szymkiewicz-Simpson overlap coefficient. We applied hierarchical clustering to the distance matrix, resulting in 28 unique pockets.

As each of these 28 unique pockets is a set of pockets present across several structures, we summarized the total number of structures in which each is found and calculated the average and maximum druggability across all structures. For each pocket in each structure, we averaged the ∆∆*G*_a_ per residue and calculated the mean residue-averaged ∆∆*G*_a_ (mean ∆∆G_a_), maximum residue-averaged ∆∆*G*_a_ (max ∆∆G_a_), and minimum ∆∆*G*_a_ (min ∆∆*G*_a_). We also calculated the OR of enrichment of each pocket in activating and inactivating mutations relative to the rest of the KD and tested its statistical significance using FET. As these metrics were very consistent across structures, we used the median across structures as the final unique pocket summary shown in Fig. 5B.

### Quantification of changes in mutational effects by regulatory domains

To quantify the changes in ∆∆*G*_a_ that result from the presence of Src regulatory domains, we fit a linear model to the ∆∆*G*_a_ dataset in full-length Src against ∆∆*G*_a_ in the KD alone and used the residuals to the fit as an estimator of ∆∆∆G_a_ (full-length minus KD). To identify mutations with significant ∆∆∆*G*_a_, we calculated *z* scores incorporating the errors of both full-length (sdFL) and KD alone (sdKD) ∆∆*G*_a_, as $Z=\frac{\text{residual}}{\frac{\text{sdFL}+\text{sdKD}}{2}}.$We defined interdomain interfaces as residues involved in direct contacts between the KD and the regulatory domains (SH3, SH2, linker) using getcontacts, and excluding water bridges. To identify spatial clusters, we selected sites with at least two mutations with ∆∆∆*G*_a_ < −1 and FDR < 0.1 and applied hierarchical clustering to a matrix with their pairwise C_α_-C_α_ distances.
