# Supplementary material for: The allosteric landscape of the Src kinase
Source: Sci Adv. 2026 Feb 11;12(7):eaea2726. doi: 10.1126/sciadv.aea2726 (PMC12893324; doi:10.1126/sciadv.aea2726)
Supplement: Supplementary file 1 — Figs. S1 to S10 Legends for movies S1 to S10 Legends for tables S1 to S3 Tables S4 to S9 [file sciadv.aea2726_sm.pdf]

Science Advances
AAAS

Supplementary Materials for

The allosteric landscape of the Src kinase

Antoni Beltran *et al.*

Corresponding author: Antoni Beltran, abeltran@ibv.csic.es; Ben Lehner, bl11@sanger.ac.uk

Sci. Adv. **12**, eaea2726 (2026)
DOI: 10.1126/sciadv.aea2726

The PDF file includes:

Figs. S1 to S10
Legends for movies S1 to S10
Legends for tables S1 to S3
Tables S4 to S9

Other Supplementary Material for this manuscript includes the following:

Movies S1 to S10
Tables S1 to S3

Supplementary Figure legends

Supplementary Figure 1: Selection assay reproducibility and thermodynamic model fitting evaluation
a. Fitness score replicate correlations for each of the 5 blocks of full-length Src kinase library, for the activity-dependent toxicity assay (top row) and sandwich abundancePCA (bottom row).
b-c. Fitness score heatmaps for Src activity (b) and abundance (c). **d**. Correlations between observed fitness values and MoCHI fitness predictions for test set variants held out in any of the 10 training folds. **e**. Percentage of explainable variance captured by 2-state, 3-state, and 4-state models.

Supplementary Figure 2: Amino acid preferences for Src kinase domain stability
a. Distributions of folding ΔΔG ($\Delta\Delta G_f$) in different secondary structure element types according to the identity of the introduced amino acid ('mutations to'). **b**. Enrichment of mutations according to the introduced amino acid ('mutations to') in the destabilizing set across the kinase domain (left panel) and as a function of secondary structure element type (right panel). Significantly enriched or depleted amino acids are labeled with a star (FDR<0.1). **c**. Distributions of $\Delta\Delta G_f$ according to the identity of the introduced amino acid ('mutations to') in specific secondary structure elements of the Src kinase.

Supplementary Figure 3: Molecular origins of loss of function in human Src.
Scatter plot comparing $\Delta\Delta G_f$ and $\Delta\Delta G_a$ across all single mutations in full-length Src. Most mutations affect either folding or activity, with a minority of mutations affecting both.

Supplementary Figure 4: Major activating allosteric sites
Src structure depicting major activatory allosteric sites and heatmap showing $\Delta\Delta G_a$ at these sites.

Supplementary Figure 5: Anisotropy in allosteric transmission across the Src KD
a. Exponential decay fits to the relationship between $|\Delta\Delta G_a|$ to the minimum heavy atom distance to the active site, for all mutations in all Src sites. $|\Delta\Delta G_a|_0$=starting $\Delta\Delta G_a$ at distance=0 (active site), k=decay rate, d=distance from the active site. Red line = exponential fit, yellow line = linear model fit, blue = running mean over a 5Å window. **b**. Exponential decay fits to the relationship between $|\Delta\Delta G_a|$ to the minimum heavy atom distance to the active site, for all mutations in Src allosteric sites and the active site. **c**. Illustrative example of a subsample of inactivating mutations ($\Delta\Delta G_a$>0) matching the distribution of effect sizes of activating mutations ($\Delta\Delta G_a$<0). **d**. Exponential decay fit to the subsample of inactivating mutations in c. **e**. Distribution of exponential decay rates (k) of the 10,000 subsamples of inactivating mutations (black), compared to the observed decay rate of activating mutations (red line). **f**. Distribution of pairwise distances between Src allosteric sites (red), compared to a null distribution calculated from random subsets of residues (n=1000, black). **g**. Distribution of median pairwise distances calculated from random subsets of residues (n=1000, black), compared to the median pairwise distance between Src allosteric sites (red vertical line). **h**. Distribution of per-site minimum distances to any other

allosteric site (red), compared to random subsets of residues (n=1000, black). **i**. Median per-site minimum distance between allosteric sites (red vertical line), compared to the distribution of medians of random subsets (n=1000, black). **j**. Exponential decay fits of $|\Delta\Delta G_a|$ with distance to the active site in the three orthogonal spatial directions, positive and negative. Decay was calculated in each spatial direction for residues located at a distance less than 10Å from the active site in the two remaining directions. **k**. Distance-corrected $\Delta\Delta G_a$ (residuals to loess smoothing curve fit, see Methods) in different secondary structure types. **l**. Exponential decay fits to the relationship between $|\Delta\Delta G_a|$ to the minimum heavy atom distance to the active site in different secondary structure types.

Supplementary Figure 6: Summary and clustering of Kinase Atlas Src surface pockets
a. Clustered matrix of Src surface pockets (defined as residues with minimum heavy atom distance< 5 Å to docked small molecules) across all structures based on the Szymkiewicz-Simpson overlap coefficient. Cluster assignments are shown as a color bar in the left side of the heatmap. **b**. Distribution of mean $\Delta\Delta G_a$ of pockets across structures for each of the 28 Src surface pockets. Each data point represents the pocket in a specific Src structure, where present. **c**. Comparison of druggability against mean $\Delta\Delta G_a$ across structures for each of the 28 Src surface pockets. Each data point represents the pocket in a specific Src structure, where present.

Supplementary Figure 7: Comparison of full-length and kinase domain alone Src activity energies
a. Fitness score replicate correlations for each of the 5 blocks of the Src kinase domain library, for the activity-dependent toxicity assay (top row) and abundancePCA (bottom row). **b**. Correlation of activity fitness measurements to in vivo phosphotyrosine levels[44]. **c**. Correlation of abundancePCA fitness measurements to in vivo Src levels[44]. **d.** Correlation between trypsin sensitivity measured as $\log_{10}(K_{50})$ and $\Delta\Delta G_f$ inferred from the aPCA data for the Src kinase domain using thermodynamic modeling. **e,f**. Heatmaps showing MoCHI inferred changes in activity free energies (d, $\Delta\Delta G_a$) and folding free energies (e, $\Delta\Delta Gf$). **g**. Heatmaps showing the changes in $\Delta\Delta G_a$ between full-length and kinase domain Src ($\Delta\Delta\Delta G_a$). **h**, Sequence and annotation of Src. Locations of individual secondary structure elements and functional regions were obtained from [50,72]. **i**. Distributions of $\Delta\Delta G_a$ in full-length and kinase domain Src. **j**. Distribution of changes in $\Delta\Delta G_a$ between full-length and kinase domain Src ($\Delta\Delta\Delta G_a$) at regulatory domain interfaces, inferred exchanging the full-length and kinase domain alone abundance datasets as the underlying folding data for MoCHI fitting. **k**. Hierarchical clustering of sites with three or more mutations with significantly more activating effects in full-length Src than in the kinase domain alone (residuals to fit>1, FDR<0.1) according to their spatial distances.

Supplementary Figure 8: Summary of allosteric pockets in full-length and kinase domain Src. Summary of the regulatory impact of Src surface pockets in a full-length and a kinase domain context, showing the odds ratios of enrichment in activating and inactivating mutations of each pocket relative to the rest of the kinase. Pockets significantly enriched or depleted (Fisher's exact test FDR<0.05) are labeled with stars.

Supplementary Figure 9: Comparison of allosteric site predictors to Src DMS data
a-b. Correlation between site-averaged $\Delta\Delta G_a$ (full-length) and allosteric coupling intensities (ACI) predicted by Ohm[62] on a residue-level, calculated using ANP as a reference point (a),

or the active site as reference point (b), using 2SRC as the reference structure. **c-d**. Comparison of allositePro[62,63] overall score (c) and perturbation score (d) with pocket-averaged $\Delta\Delta G_a$. **e**. Comparison of PASSer[64] scores with pocket-averaged $\Delta\Delta G_a$. **f**. Comparison of apop[65] scores with pocket-averaged $\Delta\Delta G_a$.

Supplementary Figure 10: Surface pockets at different clustering thresholds. Each row corresponds to a pocket at a threshold of h/2, resulting in 28 unique pockets. Some pockets become merged at more stringent thresholds as depicted by solid boxes in the table. ov = overlap coefficient (number of overlapping residues divided by total unique residues). In the case of mergers involving more than two pockets the average overlap coefficient is shown.

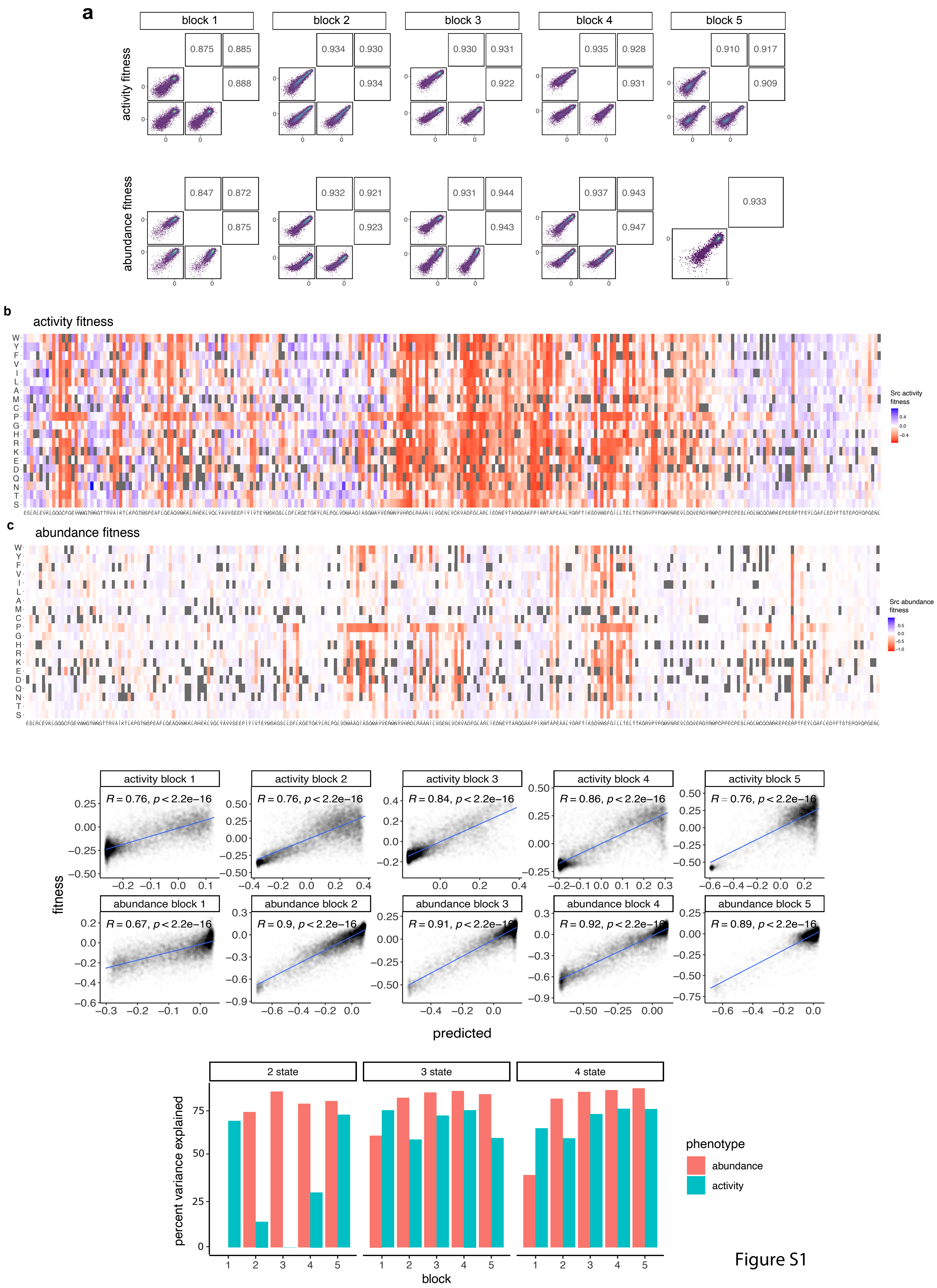
a
block 1
block 2
block 3
block 4
block 5
activity fitness
abundance fitness
0.875 0.885 0.888
0.934 0.930 0.934
0.930 0.931 0.922
0.935 0.928 0.931
0.910 0.917 0.909
0.847 0.872 0.875
0.932 0.921 0.923
0.931 0.944 0.943
0.937 0.943 0.947
0.933
b
activity fitness
Src activity fitness
c
abundance fitness
Src abundance fitness
activity block 1
R = 0.76, p < 2.2e−16
activity block 2
R = 0.76, p < 2.2e−16
activity block 3
R = 0.84, p < 2.2e−16
activity block 4
R = 0.86, p < 2.2e−16
activity block 5
R = 0.76, p < 2.2e−16
abundance block 1
R = 0.67, p < 2.2e−16
abundance block 2
R = 0.9, p < 2.2e−16
abundance block 3
R = 0.91, p < 2.2e−16
abundance block 4
R = 0.92, p < 2.2e−16
abundance block 5
R = 0.89, p < 2.2e−16
fitness
predicted
2 state
3 state
4 state
percent variance explained
block
phenotype
abundance
activity

Figure S1

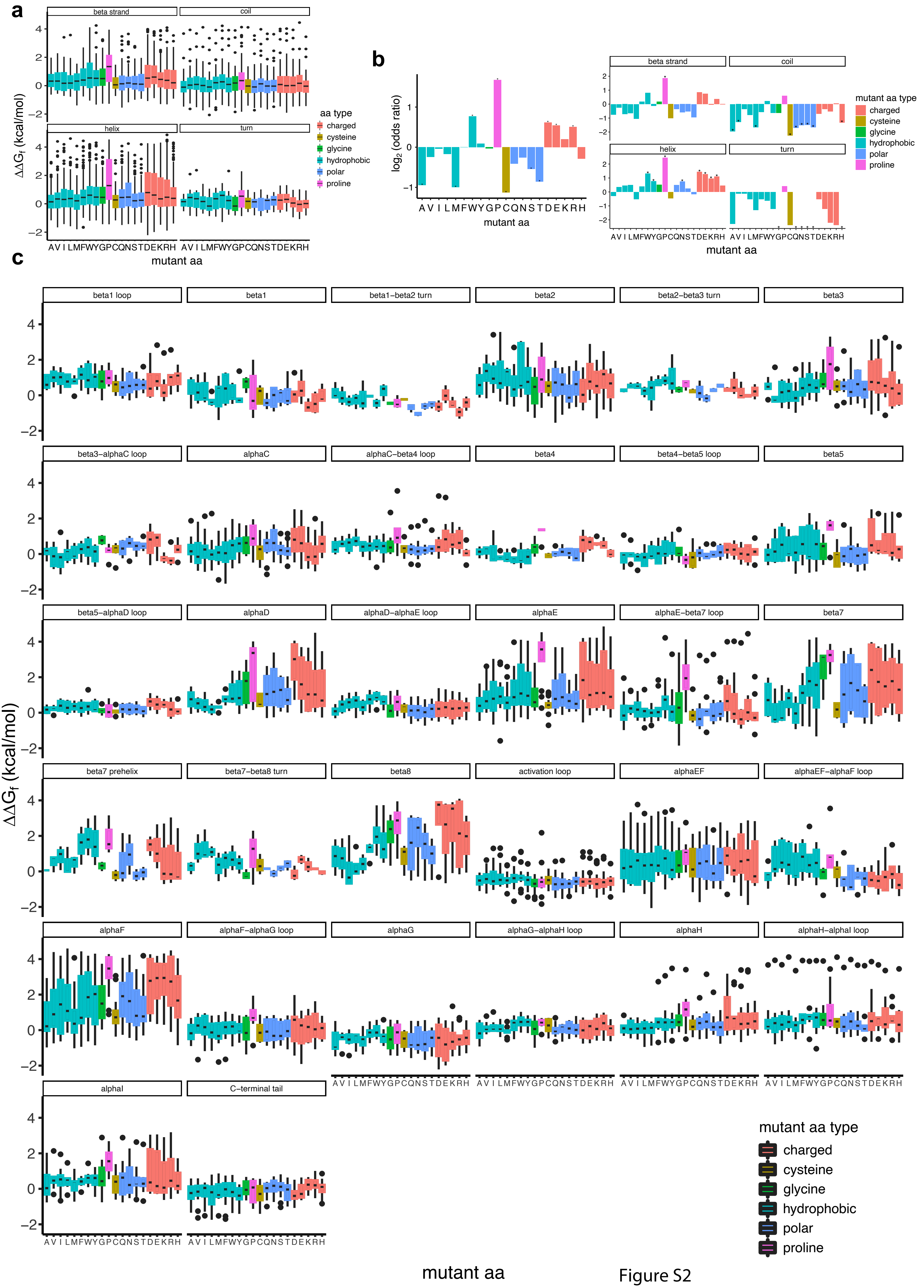
a
beta strand
coil
helix
turn
ΔΔG_f (kcal/mol)
aa type
charged
cysteine
glycine
hydrophobic
polar
proline
mutant aa
b
log2 (odds ratio)
mutant aa
mutant aa type
c
beta1 loop
beta1
beta1−beta2 turn
beta2
beta2−beta3 turn
beta3
beta3−alphaC loop
alphaC
alphaC−beta4 loop
beta4
beta4−beta5 loop
beta5
beta5−alphaD loop
alphaD
alphaD−alphaE loop
alphaE
alphaE−beta7 loop
beta7
beta7 prehelix
beta7−beta8 turn
beta8
activation loop
alphaEF
alphaEF−alphaF loop
alphaF
alphaF−alphaG loop
alphaG
alphaG−alphaH loop
alphaH
alphaH−alphaI loop
alphaI
C−terminal tail
A V I L M F W Y G P C Q N S T D E K R H
mutant aa

Figure S2

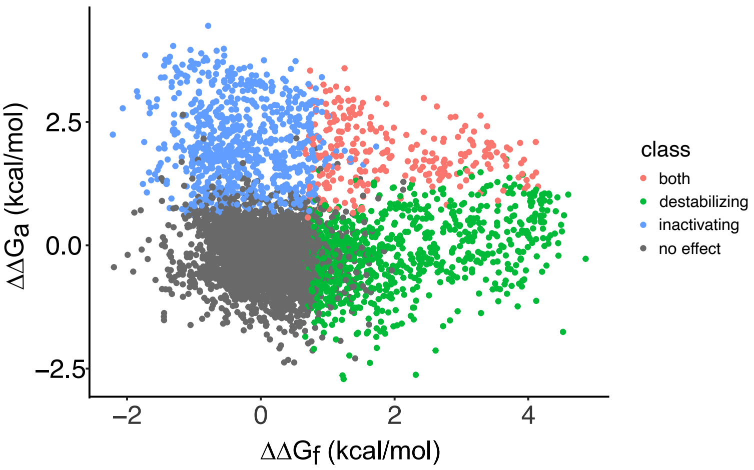

ΔΔGa (kcal/mol)
2.5
0.0
−2.5
−2
0
2
4
ΔΔGf (kcal/mol)
class
both
destabilizing
inactivating
no effect

Figure S3

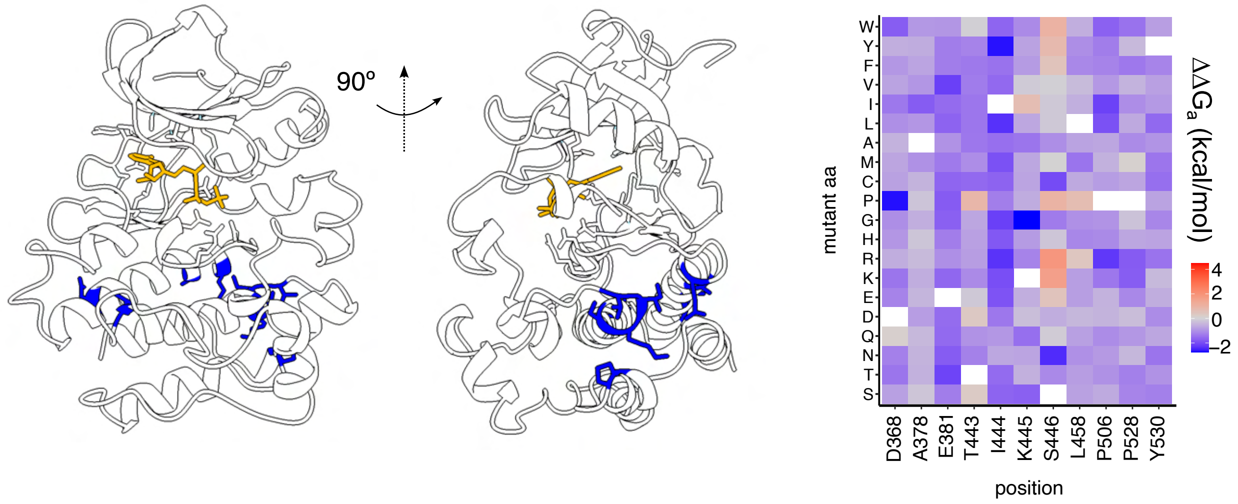
90°
W
Y
F
V
I
L
A
M
C
P
G
H
R
K
E
D
Q
N
T
S
mutant aa
D368
A378
E381
T443
I444
K445
S446
L458
P506
P528
Y530
position
ΔΔGa (kcal/mol)
4
2
0
−2

Figure S4

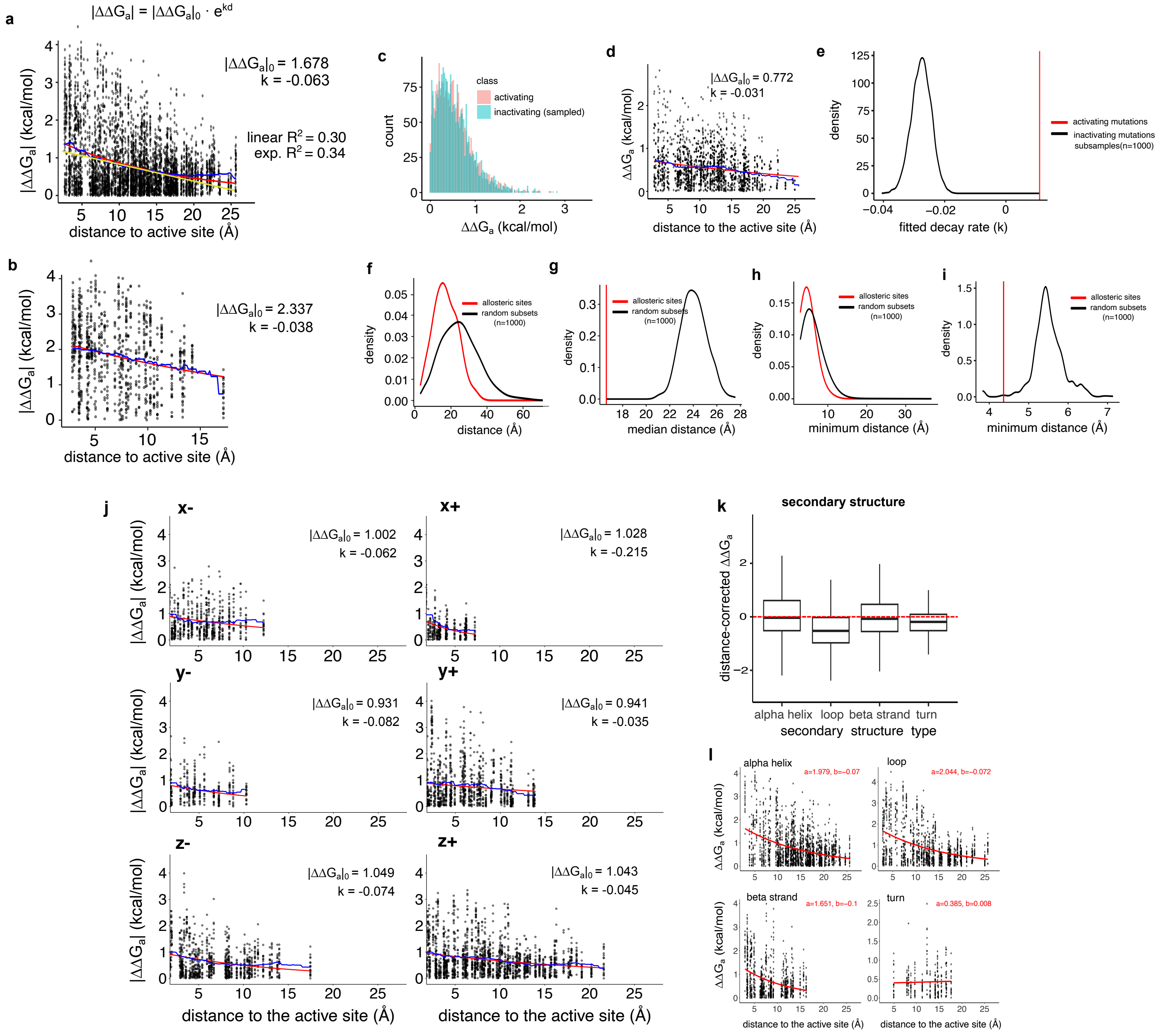
a
|ΔΔGa| = |ΔΔGa|0 · e^kd
|ΔΔGa|0 = 1.678
k = -0.063
linear R2 = 0.30
exp. R2 = 0.34
|ΔΔGa| (kcal/mol)
distance to active site (Å)
b
|ΔΔGa|0 = 2.337
k = -0.038
|ΔΔGa| (kcal/mol)
distance to active site (Å)
c
class
activating
inactivating (sampled)
count
ΔΔGa (kcal/mol)
d
|ΔΔGa|0 = 0.772
k = -0.031
ΔΔGa (kcal/mol)
distance to the active site (Å)
e
density
activating mutations
inactivating mutations subsamples(n=1000)
fitted decay rate (k)
f
allosteric sites
random subsets (n=1000)
density
distance (Å)
g
allosteric sites
random subsets (n=1000)
density
median distance (Å)
h
allosteric sites
random subsets (n=1000)
density
minimum distance (Å)
i
allosteric sites
random subsets (n=1000)
density
minimum distance (Å)
j
x-
|ΔΔGa|0 = 1.002
k = -0.062
x+
|ΔΔGa|0 = 1.028
k = -0.215
y-
|ΔΔGa|0 = 0.931
k = -0.082
y+
|ΔΔGa|0 = 0.941
k = -0.035
z-
|ΔΔGa|0 = 1.049
k = -0.074
z+
|ΔΔGa|0 = 1.043
k = -0.045
|ΔΔGa| (kcal/mol)
distance to the active site (Å)
k
secondary structure
distance-corrected ΔΔGa
alpha helix
loop
beta strand
turn
secondary structure type
l
alpha helix
a=1.979, b=-0.07
loop
a=2.044, b=-0.072
beta strand
a=1.651, b=-0.1
turn
a=0.385, b=0.008
ΔΔGa (kcal/mol)
distance to the active site (Å)

Figure S5

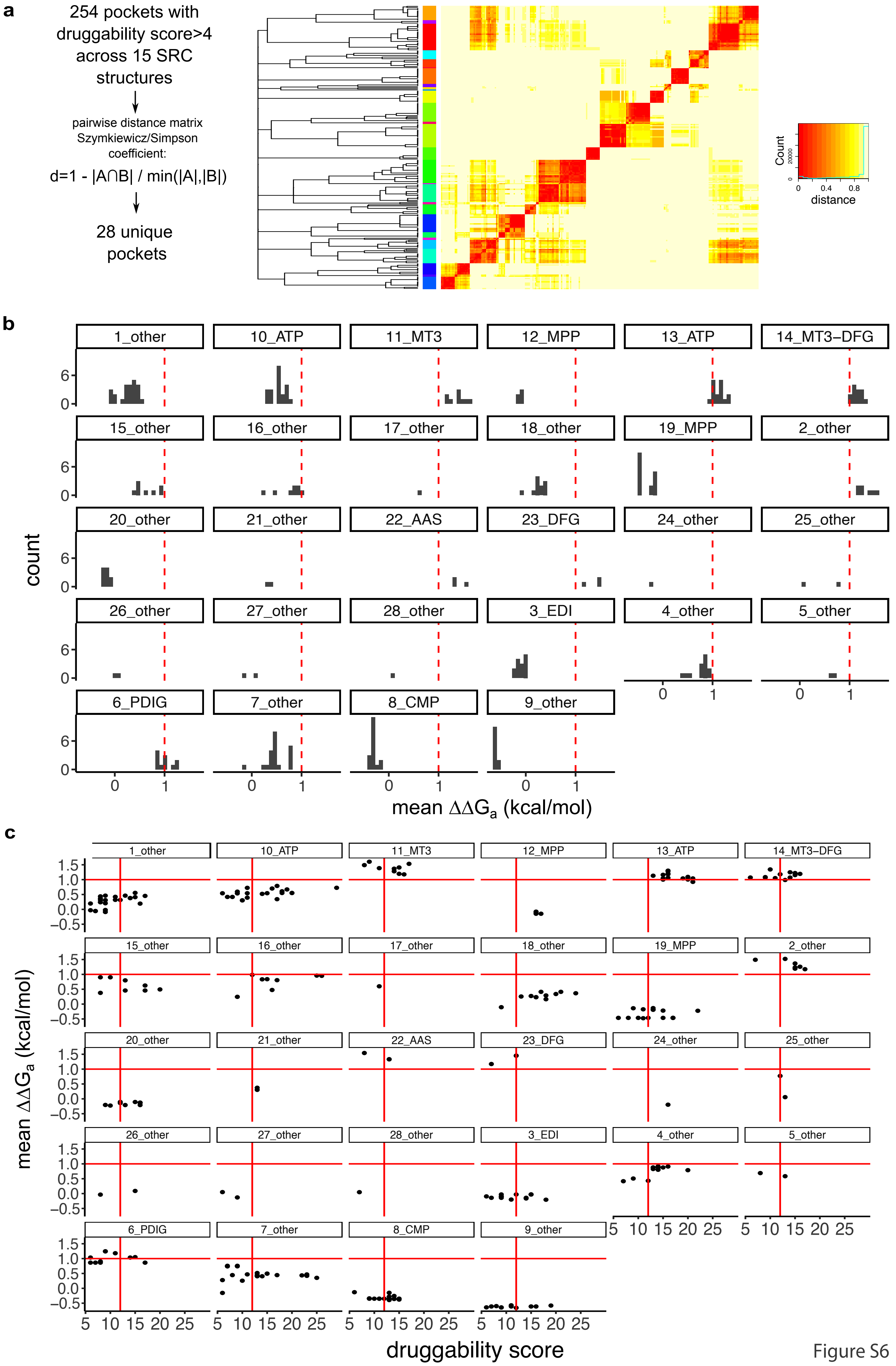
a
254 pockets with druggability score>4 across 15 SRC structures
pairwise distance matrix Szymkiewicz/Simpson coefficient:
d=1 - |A∩B| / min(|A|,|B|)
28 unique pockets
Count
distance
b
1_other
10_ATP
11_MT3
12_MPP
13_ATP
14_MT3−DFG
15_other
16_other
17_other
18_other
19_MPP
2_other
20_other
21_other
22_AAS
23_DFG
24_other
25_other
26_other
27_other
28_other
3_EDI
4_other
5_other
6_PDIG
7_other
8_CMP
9_other
count
mean ΔΔGa (kcal/mol)
c
mean ΔΔGa (kcal/mol)
druggability score

Figure S6

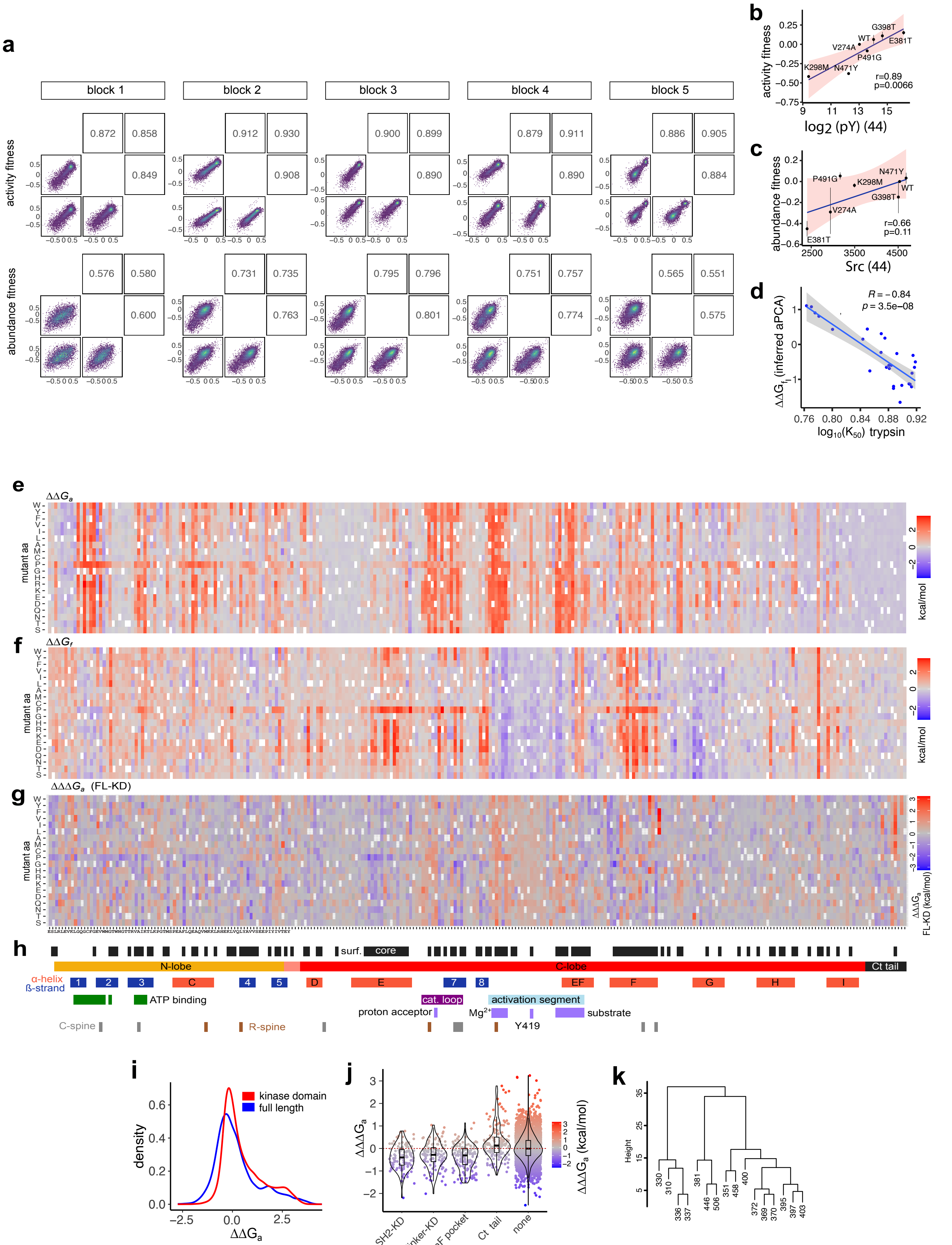
a
block 1
block 2
block 3
block 4
block 5
activity fitness
abundance fitness
0.872
0.858
0.849
0.912
0.930
0.908
0.900
0.899
0.890
0.879
0.911
0.890
0.886
0.905
0.884
0.576
0.580
0.600
0.731
0.735
0.763
0.795
0.796
0.801
0.751
0.757
0.774
0.565
0.551
0.575
b
activity fitness
log2 (pY) (44)
G398T
WT
E381T
V274A
P491G
K298M
N471Y
r=0.89
p=0.0066
c
abundance fitness
Src (44)
P491G
N471Y
K298M
WT
G398T
V274A
E381T
r=0.66
p=0.11
d
ΔΔGf (inferred aPCA)
log10(K50) trypsin
R = −0.84
p = 3.5e−08
e
ΔΔGa
mutant aa
kcal/mol
f
ΔΔGf
g
ΔΔΔGa (FL-KD)
ΔΔΔGa FL-KD (kcal/mol)
h
surf.
core
N-lobe
C-lobe
Ct tail
α-helix
ß-strand
ATP binding
cat. loop
activation segment
proton acceptor
Mg2+
substrate
C-spine
R-spine
Y419
i
density
ΔΔGa
kinase domain
full length
j
ΔΔΔGa
ΔΔΔGa (kcal/mol)
SH2-KD
SH3-linker-KD
aF pocket
Ct tail
none
k
Height

Figure S7

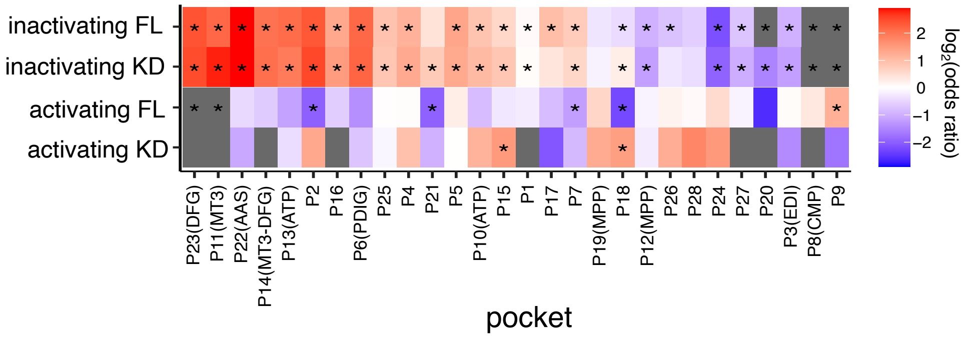
inactivating FL
inactivating KD
activating FL
activating KD
P23(DFG)
P11(MT3)
P22(AAS)
P14(MT3-DFG)
P13(ATP)
P2
P16
P6(PDIG)
P25
P4
P21
P5
P10(ATP)
P15
P1
P17
P7
P19(MPP)
P18
P12(MPP)
P26
P28
P24
P27
P20
P3(EDI)
P8(CMP)
P9
pocket
2
1
0
−1
−2
log2(odds ratio)

Figure S8

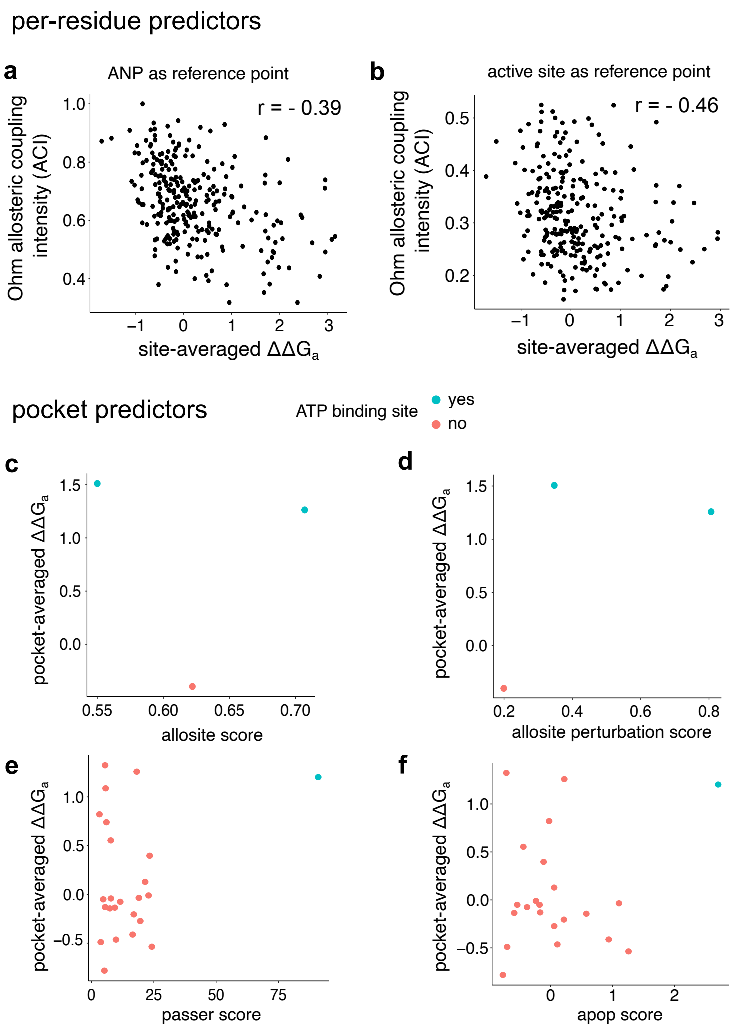
per-residue predictors
a
ANP as reference point
r = - 0.39
Ohm allosteric coupling intensity (ACI)
site-averaged ΔΔG$_a$
b
active site as reference point
r = - 0.46
Ohm allosteric coupling intensity (ACI)
site-averaged ΔΔG$_a$
pocket predictors
ATP binding site
yes
no
c
pocket-averaged ΔΔG$_a$
allosite score
d
pocket-averaged ΔΔG$_a$
allosite perturbation score
e
pocket-averaged ΔΔG$_a$
passer score
f
pocket-averaged ΔΔG$_a$
apop score

Figure S9

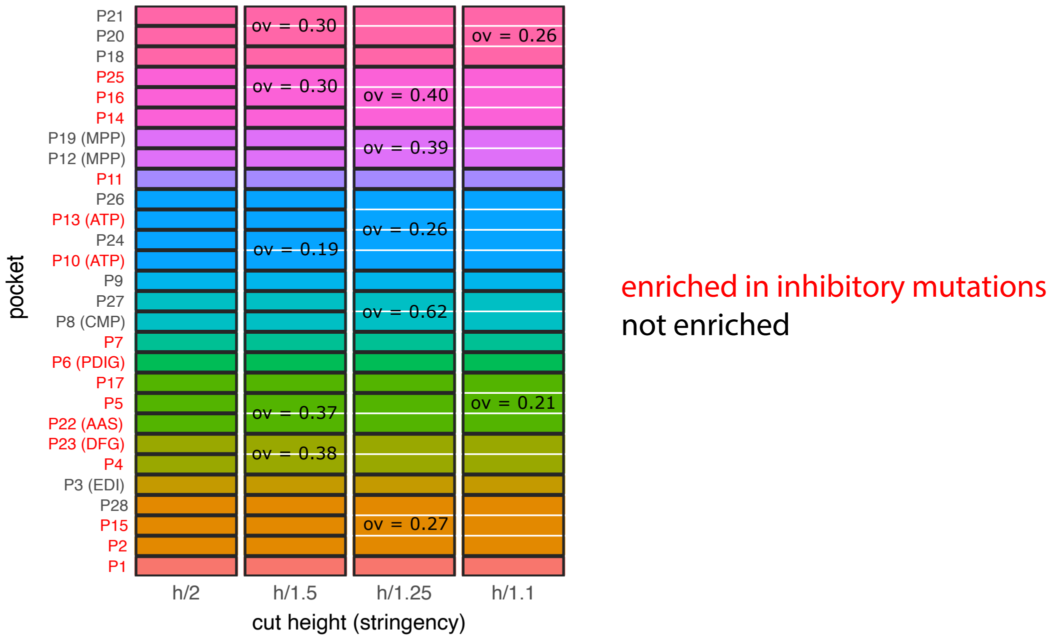
P21
P20
P18
P25
P16
P14
P19 (MPP)
P12 (MPP)
P11
P26
P13 (ATP)
P24
P10 (ATP)
P9
P27
P8 (CMP)
P7
P6 (PDIG)
P17
P5
P22 (AAS)
P23 (DFG)
P4
P3 (EDI)
P28
P15
P2
P1
pocket
ov = 0.30
ov = 0.30
ov = 0.19
ov = 0.37
ov = 0.38
ov = 0.40
ov = 0.39
ov = 0.26
ov = 0.62
ov = 0.27
ov = 0.26
ov = 0.21
h/2
h/1.5
h/1.25
h/1.1
cut height (stringency)
enriched in inhibitory mutations
not enriched

Figure S10

Supplementary Movies

Supplementary Movie 1. Average mutation effects on stability in full-length Src. The average $\Delta\Delta G_f$ of the 19 substitutions in each residue in full-length Src is represented in the colour scale. PDB ID: 2SRC.

Supplementary Movie 2. Average mutation effects on activity in full-length Src. The average $\Delta\Delta G_a$ of the 19 substitutions in each residue in full-length Src is represented in the colour scale. PDB ID: 2SRC.

Supplementary Movie 3. The landscape of activity-modulating mutations in full-length Src. The $\log_2$ odds ratio of enrichment in mutations modulating full-length Src activity ($|\Delta\Delta G_a|>0.5$, FDR<0.1, z-test) is represented in the colour scale. PDB ID: 2SRC.

Supplementary Movie 4. Average mutation effects on activity in the Src surface (full-length dataset). The average $\Delta\Delta G_a$ of the 19 substitutions in each residue in full-length Src is represented in the colour scale. PDB ID: 2SRC.

Supplementary Movie 5. Maximum mutation effects on activity in the Src surface (full-length dataset). The maximum $\Delta\Delta G_f$ of all 19 substitutions in each residue in full-length Src is represented in the colour scale. PDB ID: 2SRC.

Supplementary Movie 6. Average mutation effects on stability in the Src kinase domain. The average $\Delta\Delta G_f$ of the 19 substitutions in each residue in the Src kinase domain is represented in the colour scale. PDB ID: 2SRC.

Supplementary Movie 7. Average mutation effects on activity in the Src kinase domain. The average $\Delta\Delta G_a$ of the 19 substitutions in each residue in the Src kinase domain is represented in the colour scale. PDB ID: 2SRC.

Supplementary Movie 8. The landscape of activity-modulating mutations in the Src kinase domain. The $\log_2$ odds ratio of enrichment in mutations modulating Src kinase domain activity ($|\Delta\Delta G_a|>0.5$, FDR<0.1, z-test) is represented in the colour scale. PDB ID: 2SRC.

Supplementary Movie 9. Average mutation effects on activity in the Src surface (kinase domain dataset). The average $\Delta\Delta G_a$ of the 19 substitutions in each residue in the Src kinase domain is represented in the colour scale. PDB ID: 2SRC.

Supplementary Movie 10. Maximum mutation effects on activity in the Src surface (kinase domain dataset). The maximum $\Delta\Delta G_f$ of all 19 substitutions in each residue in the Src kinase domain is represented in the colour scale. PDB ID: 2SRC.

Supplementary tables

Supplementary table 1: Fitness scores and errors
Supplementary table 2: Fitted values of $\Delta\Delta G_f$ and $\Delta\Delta G_a$
Supplementary table 3: Src surface pocket summary

Supplementary table 4: Plasmids

pGJJ133	aPCA empty	Available upon request (Material Transfer Agreement required)
pTB022	toxicity empty	https://benchling.com/s/seq-rGJbDMKRao9IdlAqhMoJ?m=slm-cUTBKgetTLCpJ3TveV5w
pTB198	sandwichPCA empty	https://benchling.com/s/seq-mve4HVJwo23HnO6BPNym?m=slm-lNfOeXfnOvLHoarUjhPK
pTB109	aPCA Src KD	https://benchling.com/s/seq-obS1wabcFstnPlwNCdPI?m=slm-an1b6yZtzRU1xIytarI5
pTB112	toxicity Src KD	https://benchling.com/s/seq-PUIubmPUQrNsuv9YkjJM?m=slm-KBT9Fq1il6zXODI1mvnj
pTB043	sandwichPCA full length	https://benchling.com/s/seq-0sA5ySViNYIlnltmMAFI?m=slm-27zYqdcFKogMjblGqR1y
pTB023	toxicity full length	https://benchling.com/s/seq-PdGjC1KRegS3IUGnMNPh?m=slm-CCYco9fv12T7nVaTxXhf

Supplementary table 5: Oligonucleotides

Amplification of SRC KD from full length gBlock	
oTB063	CAATATGCTAGCGATGCTTGGGAGATCCCTC
oTB064	TAATTTAAAGCTTCAAGTTCTCTC
Introduction of start codon in pTB112	
oTB214	atgGATGCTTGGGAGATCCCTC
oTB215	GCTAGCCTCCTTGACGTT
oPool and backbone amplification for Gibson assembly	
oTB447_b1_ins_F	GATGCTTGGGAGATCCCTC
oTB448_b1_ins_R	CACTCACAACTGCATACAATTG
oTB449_b2_ins_F	AGCACAAGTCATGAAGAAGC
oTB450_b2_ins_R	CACGGTGTACGTAATTCATTC
oTB451_b3_ins_F	CATGGCCGCCCAGATTG
oTB452_b3_ins_R	CGTGAACCTTCCATATAAGGC
oTB453_b4_ins_F	GTGCAAAGTTCCCCATCAAG
oTB454_b4_ins_R	CATAAGGTCGTGCAAGCTC
oTB455_b5_ins_F	GAACGTGGTTATAGAATGCC
oTB220_b5_ins_R	GCGTGACATAACTAATTTAAAGC
oTB457_b1_bb_F	CAATTGTATGCAGTTGTGAGTG
oTB458_b1_bb_R	GAGGGATCTCCCAAGCATC
oTB459_b2_bb_F	GAATGAATTACGTACACCGTG

oTB460_b2_bb_R	AGCTTCTTCATGACTTGTGC
oTB461_b3_bb_F	GCCTTATATGGAAGGTTCACG
oTB462_b3_bb_R	CAATCTGGGCGGCCATG
oTB463_b4_bb_F	GAGCTTGCACGACCTTATG
oTB464_b4_bb_R	CTTGATGGGGAACTTTGCAC
oTB241_b5_bb_F	AAGCTTTAAATTAGTTATGTCACG
oTB466_b5_bb_R	GGCATTCTATAACCACGTTC
qPCR quantification oligos	
oGJJ152	GCCTACATACCTCGCTCTGC
oGJJ153	CAACCCGGTAAGACACGACT
Frameshifting PCR1 oligos (aPCA)	
oTB302_b1_fs_F	ACACTCTTTCCCTACACGACGCTCTTCCGATCTATGCTTGGGAGATCCCTC
oTB303_302_+1	ACACTCTTTCCCTACACGACGCTCTTCCGATCTNATGCTTGGGAGATCCCTC
oTB304_302_+2	ACACTCTTTCCCTACACGACGCTCTTCCGATCTNCATGCTTGGGAGATCCCTC
oTB305_302_+3	ACACTCTTTCCCTACACGACGCTCTTCCGATCTGGCATGCTTGGGAGATCCCTC
oTB306_302_+4	ACACTCTTTCCCTACACGACGCTCTTCCGATCTCTGNATGCTTGGGAGATCCCTC
oTB307_302_+5	ACACTCTTTCCCTACACGACGCTCTTCCGATCTNWWANATGCTTGGGAGATCCCTC
oTB308_b1_fs_R	GTGACTGGAGTTCAGACGTGTGCTCTTCCGATCTCACTCACAACTGCATACAATTG
oTB309_308_+1	GTGACTGGAGTTCAGACGTGTGCTCTTCCGATCTGCACTCACAACTGCATACAATTG
oTB310_308_+2	GTGACTGGAGTTCAGACGTGTGCTCTTCCGATCTAGCACTCACAACTGCATACAATTG
oTB311_308_+3	GTGACTGGAGTTCAGACGTGTGCTCTTCCGATCTTAGCACTCACAACTGCATACAATTG
oTB312_308_+4	GTGACTGGAGTTCAGACGTGTGCTCTTCCGATCTWTTGCACTCACAACTGCATACAATTG
oTB313_308_+5	GTGACTGGAGTTCAGACGTGTGCTCTTCCGATCTSCATGCACTCACAACTGCATACAATTG
oTB471_449_b2_FS_F	ACACTCTTTCCCTACACGACGCTCTTCCGATCTAGCACAAGTCATGAAGAAGC
oTB472_449_+1	ACACTCTTTCCCTACACGACGCTCTTCCGATCTTAGCACAAGTCATGAAGAAGC
oTB473_449_+2	ACACTCTTTCCCTACACGACGCTCTTCCGATCTCTAGCACAAGTCATGAAGAAGC
oTB474_449_+3	ACACTCTTTCCCTACACGACGCTCTTCCGATCTGCTAGCACAAGTCATGAAGAAGC
oTB475_449_+4	ACACTCTTTCCCTACACGACGCTCTTCCGATCTNNNTAGCACAAGTCATGAAGAAGC

oTB476_449+5	ACACTCTTTCCCTACACGACGCTCTTCCGATCTNNNTTAGCACAAGTCATGAAGAAGC
oTB477_450_b2_FS_R	GTGACTGGAGTTCAGACGTGTGCTCTTCCGATCTCACGGTGTACGTAATTCATTC
oTB478_450_+1	GTGACTGGAGTTCAGACGTGTGCTCTTCCGATCTGCACGGTGTACGTAATTCATTC
oTB479_450_+2	GTGACTGGAGTTCAGACGTGTGCTCTTCCGATCTTGCACGGTGTACGTAATTCATTC
oTB480_450_+3	GTGACTGGAGTTCAGACGTGTGCTCTTCCGATCTATGCACGGTGTACGTAATTCATTC
oTB481_450_+4	GTGACTGGAGTTCAGACGTGTGCTCTTCCGATCTWSTTCACGGTGTACGTAATTCATTC
oTB482_450_+5	GTGACTGGAGTTCAGACGTGTGCTCTTCCGATCTSWNWWCACGGTGTACGTAATTCATTC
oTB483_451_b3_FS_F	ACACTCTTTCCCTACACGACGCTCTTCCGATCTCATGGCCGCCCAGATTG
oTB484_451_+1	ACACTCTTTCCCTACACGACGCTCTTCCGATCTGCATGGCCGCCCAGATTG
oTB485_451_+2	ACACTCTTTCCCTACACGACGCTCTTCCGATCTAGCATGGCCGCCCAGATTG
oTB486_451_+3	ACACTCTTTCCCTACACGACGCTCTTCCGATCTTTGCATGGCCGCCCAGATTG
oTB487_451_+4	ACACTCTTTCCCTACACGACGCTCTTCCGATCTNNNNCATGGCCGCCCAGATTG
oTB488_451_+5	ACACTCTTTCCCTACACGACGCTCTTCCGATCTNNNNWCATGGCCGCCCAGATTG
oTB489_452_b3_FS_R	GTGACTGGAGTTCAGACGTGTGCTCTTCCGATCTCGTGAACCTTCCATATAAGGC
oTB490_452_+1	GTGACTGGAGTTCAGACGTGTGCTCTTCCGATCTACGTGAACCTTCCATATAAGGC
oTB491_452_+2	GTGACTGGAGTTCAGACGTGTGCTCTTCCGATCTGACGTGAACCTTCCATATAAGGC
oTB492_452_+3	GTGACTGGAGTTCAGACGTGTGCTCTTCCGATCTTTACGTGAACCTTCCATATAAGGC
oTB493_452_+4	GTGACTGGAGTTCAGACGTGTGCTCTTCCGATCTWSSACGTGAACCTTCCATATAAGGC
oTB494_452_+5	GTGACTGGAGTTCAGACGTGTGCTCTTCCGATCTSWWWNCGTGAACCTTCCATATAAGGC
oTB495_453_b4_FS_F	ACACTCTTTCCCTACACGACGCTCTTCCGATCTGTGCAAAGTTCCCCATCAAG
oTB496_453_+1	ACACTCTTTCCCTACACGACGCTCTTCCGATCTAGTGCAAAGTTCCCCATCAAG
oTB497_453_+2	ACACTCTTTCCCTACACGACGCTCTTCCGATCTTAGTGCAAAGTTCCCCATCAAG
oTB498_453_+3	ACACTCTTTCCCTACACGACGCTCTTCCGATCTCCCGTGCAAAGTTCCCCATCAAG
oTB499_453_+4	ACACTCTTTCCCTACACGACGCTCTTCCGATCTNNAAGTGCAA

	AGTTCCCCATCAAG
oTB500_453_+5	ACACTCTTTCCCTACACGACGCTCTTCCGATCTNNNNNGTGCAAAGTTCCCCATCAAG
oTB501_454_b4_FS_R	GTGACTGGAGTTCAGACGTGTGCTCTTCCGATCTCATAAGGTCGTGCAAGCTC
oTB502_454_+1	GTGACTGGAGTTCAGACGTGTGCTCTTCCGATCTGCATAAGGTCGTGCAAGCTC
oTB503_454_+2	GTGACTGGAGTTCAGACGTGTGCTCTTCCGATCTTGCATAAGGTCGTGCAAGCTC
oTB504_454_+3	GTGACTGGAGTTCAGACGTGTGCTCTTCCGATCTATGCATAAGGTCGTGCAAGCTC
oTB505_454_+4	GTGACTGGAGTTCAGACGTGTGCTCTTCCGATCTNNNGCATAAGGTCGTGCAAGCTC
oTB506_454_+5	GTGACTGGAGTTCAGACGTGTGCTCTTCCGATCTNNNSGCATAAGGTCGTGCAAGCTC
oTB507_455_b5_FS_F	ACACTCTTTCCCTACACGACGCTCTTCCGATCTGAACGTGGTTATAGAATGCC
oTB508_455_+1	ACACTCTTTCCCTACACGACGCTCTTCCGATCTTGAACGTGGTTATAGAATGCC
oTB509_455_+2	ACACTCTTTCCCTACACGACGCTCTTCCGATCTCTGAACGTGGTTATAGAATGCC
oTB510_455_+3	ACACTCTTTCCCTACACGACGCTCTTCCGATCTACTGAACGTGGTTATAGAATGCC
oTB511_455_+4	ACACTCTTTCCCTACACGACGCTCTTCCGATCTNNCTGAACGTGGTTATAGAATGCC
oTB512_455_+5	ACACTCTTTCCCTACACGACGCTCTTCCGATCTNNNNTGAACGTGGTTATAGAATGCC
oGJJ589_b5_FS_R	GTGACTGGAGTTCAGACGTGTGCTCTTCCGATCTGCGTGACATAACTAATTTAAAGC
oGJJ590_589_+1	GTGACTGGAGTTCAGACGTGTGCTCTTCCGATCTNGCGTGACATAACTAATTTAAAGC
oGJJ591_589_+2	GTGACTGGAGTTCAGACGTGTGCTCTTCCGATCTNNGCGTGACATAACTAATTTAAAGC
oGJJ592_589_+3	GTGACTGGAGTTCAGACGTGTGCTCTTCCGATCTHNHGCGTGACATAACTAATTTAAAGC
oGJJ593_589_+4	GTGACTGGAGTTCAGACGTGTGCTCTTCCGATCTHWWHGCGTGACATAACTAATTTAAAGC
oGJJ594_589_+5	GTGACTGGAGTTCAGACGTGTGCTCTTCCGATCTHWWAAGCGTGACATAACTAATTTAAAGC
oTB513_470+	GTGACTGGAGTTCAGACGTGTGCTCTTCCGATCTCCCACCACCTCCactAAG
oTB514_470_+1	GTGACTGGAGTTCAGACGTGTGCTCTTCCGATCTATCCCACCACCTCCactAAG
oTB515_470_+2	GTGACTGGAGTTCAGACGTGTGCTCTTCCGATCTTGACCCACCACCTCCactAAG
oTB516_470_+3	GTGACTGGAGTTCAGACGTGTGCTCTTCCGATCTGAGTCCCACCACCTCCactAAG

oTB517_470_+4	GTGACTGGAGTTCAGACGTGTGCTCTTCCGATCTSWTGDCCC ACCACCTCCactAAG
oTB518_470_+5	GTGACTGGAGTTCAGACGTGTGCTCTTCCGATCTWSNNDCCC ACCACCTCCactAAG
Frameshifting PCR1 oligos (mRNA display)	
o_093_SRC_F	ACACTCTTTCCCTACACGACGCTCTTCCGATCTNNCCGCCCAG ATTGCATCC
o_095_SRC_F	ACACTCTTTCCCTACACGACGCTCTTCCGATCTNWNCCGCCC AGATTGCATCC
o_097_SRC_F	ACACTCTTTCCCTACACGACGCTCTTCCGATCTNNHNCCGCCC AGATTGCATCC
o_099_SRC_F	ACACTCTTTCCCTACACGACGCTCTTCCGATCTNHNCCGCCCA GATTGCATCC
o_101_SRC_F	ACACTCTTTCCCTACACGACGCTCTTCCGATCTNHDNCCGCCC AGATTGCATCC
o_103_SRC_F	ACACTCTTTCCCTACACGACGCTCTTCCGATCTNDDNCCGCCC AGATTGCATCC
o_092_SRC_R	GTGACTGGAGTTCAGACGTGTGCTCTTCCGATCTNHDNTGAA CCTTCCATATAAGGCAGC
o_094_SRC_R	GTGACTGGAGTTCAGACGTGTGCTCTTCCGATCTNDNTGAAC CTTCCATATAAGGCAGC
o_096_SRC_R	GTGACTGGAGTTCAGACGTGTGCTCTTCCGATCTNDHNTGAA CCTTCCATATAAGGCAGC
o_098_SRC_R	GTGACTGGAGTTCAGACGTGTGCTCTTCCGATCTNHWDNTGA ACCTTCCATATAAGGCAGC
o_0100_SRC_R	GTGACTGGAGTTCAGACGTGTGCTCTTCCGATCTNDNDHNTG AACCTTCCATATAAGGCAGC
o_0102_SRC_R	GTGACTGGAGTTCAGACGTGTGCTCTTCCGATCTNNDNDNTG AACCTTCCATATAAGGCAGC

Supplementary Table 6: human Src sequences

Src kinase domain:
CTAGCGATGCTTGGGAGATCCCTCGTGAATCACTGCGTCTTGAGGTAAAGTTAGGCCAGGGATGC
TTTGGGGAGGTGTGGATGGGCACGTGGAACGGTACTACCAGGGTTGCAATTAAGACTCTGAAAC
CCGGAACCATGTCTCCTGAGGCGTTCCTGCAAGAAGCACAAGTCATGAAGAAGCTACGTCATGA
GAAGCTAGTGCAATTGTATGCAGTTGTGAGTGAAGAGCCGATCTACATTGTCACTGAGTACATGAG
CAAGGGTTCTTTGCTGGACTTCTTGAAGGGTGAAACCGGCAAATACCTGAGACTTCCCCAGTTGG
TAGACATGGCCGCCCAGATTGCATCCGGTATGGCTTACGTGGAGAGAATGAATTACGTACACCGT
GATCTAAGAGCTGCGAACATACTGGTTGGAGAAAACTTGGTATGTAAGGTCGCTGATTTCGGTCTG
GCGAGGCTTATTGAAGACAATGAATACACTGCACGTCAAGGTGCAAAGTTCCCCATCAAGTGGAC
GGCTCCAGAGGCTGCCTTATATGGAAGGTTCACGATAAAGTCCGATGTGTGGAGTTTCGGGATAT
TGTTAACAGAATTGACAACGAAAGGACGTGTACCATATCCTGGCATGGTTAATAGAGAAGTACTTG
ACCAGGTAGAACGTGGTTATAGAATGCCATGCCCTCCGGAGTGTCCCGAGAGCTTGCACGACCTT
ATGTGTCAGTGTTGGAGGAAAGAGCCTGAGGAGAGGCCTACATTCGAGTATCTACAAGCATTCTT
AGAAGACTACTTCACGTCCACAGAACCACAGTACCAACCCGGAGAGAACTTGA

Full length Src:

ATGGGCAGCAATAAGTCAAAGCCGAAGGATGCAAGCCAAAGGCGTAGGTCTTTGGAGCCTGCCG
AGAATGTACATGGAGCTGGTGGTGGAGCTTTTCCGGCCAGCCAGACGCCCTCCAAACCCGCGTC
TGCTGATGGTCACCGTGGGCCAAGTGCTGCTTTTGCGCCCGCTGCAGCGGAGCCTAAGCTATTC
GGGGGTTTTAACAGTAGCGACACCGTAACGAGCCCGCAGAGAGCAGGTCCGTTGGCAGGGGGC
GTGACTACGTTCGTCGCCCTATACGACTACGAGTCTAGGACAGAGACTGACTTGAGCTTCAAAAA
GGGAGAACGTCTGCAGATCGTAAACAATACAGAGGGTGACTGGTGGCTTGCACATTCTCTTAGTA
CTGGGCAGACAGGTTATATTCCGAGCAACTATGTCGCACCGAGTGATTCAATACAGGCAGAAGAG
TGGTATTTTGGAAAAATTACTCGTAGGGAGTCCGAGAGATTATTGCTTAACGCAGAGAACCCTCGT
GGGACGTTTCTGGTCAGGGAAAGCGAAACAACAAAAGGAGCGTACTGCTTAAGCGTAAGCGATT
TCGACAATGCCAAAGGTCTTAACGTTAAGCATTATAAGATTAGGAAGTTGGACTCCGGGGGCTTTT
ATATAACGAGCAGAACCCAATTTAACTCTCTACAGCAATTGGTTGCATATTACTCCAAACATGCAGA
CGGTCTATGTCATCGTTTGACAACTGTTTGCCCCACAAGTAAGCCTCAGACGCAAGGTTTAGCAA
AGGATGCTTGGGAGATCCCTCGTGAATCACTGCGTCTTGAGGTAAAGTTAGGCCAGGGATGCTTT
GGGGAGGTGTGGATGGGCACGTGGAACGGTACTACCAGGGTTGCAATTAAGACTCTGAAACCCG
GAACCATGTCTCCTGAGGCGTTCCTGCAAGAAGCACAAGTCATGAAGAAGCTACGTCATGAGAAG
CTAGTGCAATTGTATGCAGTTGTGAGTGAAGAGCCGATCTACATTGTCACTGAGTACATGAGCAAG
GGTTCTTTGCTGGACTTCTTGAAGGGTGAAACCGGCAAATACCTGAGACTTCCCCAGTTGGTAGA
CATGGCCGCCCAGATTGCATCCGGTATGGCTTACGTGGAGAGAATGAATTACGTACACCGTGATC
TAAGAGCTGCGAACATACTGGTTGGAGAAAACTTGGTATGTAAGGTCGCTGATTTCGGTCTGGCG
AGGCTTATTGAAGACAATGAATACACTGCACGTCAAGGTGCAAAGTTCCCCATCAAGTGGACGGC
TCCAGAGGCTGCCTTATATGGAAGGTTCACGATAAAGTCCGATGTGTGGAGTTTCGGGATATTGTT
AACAGAATTGACAACGAAAGGACGTGTACCATATCCTGGCATGGTTAATAGAGAAGTACTTGACCA
GGTAGAACGTGGTTATAGAATGCCATGCCCTCCGGAGTGTCCCGAGAGCTTGCACGACCTTATGT
GTCAGTGTTGGAGGAAAGAGCCTGAGGAGAGGCCTACATTCGAGTATCTACAAGCATTCTTAGAA
GACTACTTCACGTCCACAGAACCACAGTACCAACCCGGAGAGAACTTGA

Supplementary Table 7: Library design

Block	start aa	end aa	Backgrounds (neutral, gain of function*, loss of function#)
1	268	326	WT, E273G*, G282A#, E283L*, G287P#, K298M#, T304A, E313R#, V316I#, E323R*
2	321	381	WT, T341I*, L328I#, K354R*, D368V*, A370L, K354P#, S345P#, G355V, M377F#
3	376	435	WT, E381K*, R382P#, R391A#, I395C, T420V*, R412F#, Y419A#, P434H, I429A#
4	431	491	WT, W431V#, L454A#, E473T*, E489S, T443W*, L458V, T443P#, I444F#, I444P
5	486	536	WT, D496Y*, E508G, Y514R, P532W, Y530D*, M498Q, F523K, Y514N#, L494P#

Library block 1

5’ constant region:

GATGCTTGGGAGATCCCTCGT
Variable region:
GAATCACTGCGTCTTGAGGTAAAGTTAGGCCAGGGATGCTTTGGGGAGGTGTGGATGGGCACGT
GGAACGGTACTACCAGGGTTGCAATTAAGACTCTGAAACCCGGAACCATGTCTCCTGAGGCGTTC
CTGCAAGAAGCACAAGTCATGAAGAAGCTACGTCATGAGAAGCTAGTG

3’ constant region:
CAATTGTATGCAGTTGTGAGTG

<u>Library block 2</u>
5’ constant region:
AGCACAAGTCATGAAGAAGCTA
Variable region:
CGTCATGAGAAGCTAGTGCAATTGTATGCAGTTGTGAGTGAAGAGCCGATCTACATTGTCACTGAG
TACATGAGCAAGGGTTCTTTGCTGGACTTCTTGAAGGGTGAAACCGGCAAATACCTGAGACTTCC
CCAGTTGGTAGACATGGCCGCCCAGATTGCATCCGGTATGGCTTACGTGGAG
3’ constant region:
AGAATGAATTACGTACACCGTG

<u>Library block 3</u>
5’ constant region:
CATGGCCGCCCAGATTGCATCC
Variable region:
GGTATGGCTTACGTGGAGAGAATGAATTACGTACACCGTGATCTAAGAGCTGCGAACATACTGGTT
GGAGAAAACTTGGTATGTAAGGTCGCTGATTTCGGTCTGGCGAGGCTTATTGAAGACAATGAATAC
ACTGCACGTCAAGGTGCAAAGTTCCCCATCAAGTGGACGGCTCCAGAG
3’ constant region:
GCTGCCTTATATGGAAGGTTCACG

<u>Library block 4</u>
5’ constant region:
GTGCAAAGTTCCCCATCAAG
Variable region:
TGGACGGCTCCAGAGGCTGCCTTATATGGAAGGTTCACGATAAAGTCCGATGTGTGGAGTTTCGG
GATATTGTTAACAGAATTGACAACGAAAGGACGTGTACCATATCCTGGCATGGTTAATAGAGAAGTA
CTTGACCAGGTAGAACGTGGTTATAGAATGCCATGCCCTCCGGAGTGTCCC

3’ constant region:
GAGAGCTTGCACGACCTTATG

<u>Library block 5</u>
5’ constant region:
GAACGTGGTTATAGAATGCCA
Variable region:
TGCCCTCCGGAGTGTCCCGAGAGCTTGCACGACCTTATGTGTCAGTGTTGGAGGAAAGAGCCTG
AGGAGAGGCCTACATTCGAGTATCTACAAGCATTCTTAGAAGACTACTTCACGTCCACAGAACCAC
AGTACCAACCCGGAGAGAACTTG
3’ constant region:
AAGCTTTAAATTAGTTATGTCACGC

Genetic background sequences:

Block 1:
>WT
GATGCTTGGGAGATCCCTCGTGAATCACTGCGTCTTGAGGTAAAGTTAGGCCAGGGATGCTTTGG
GGAGGTGTGGATGGGCACGTGGAACGGTACTACCAGGGTTGCAATTAAGACTCTGAAACCCGGA
ACCATGTCTCCTGAGGCGTTCCTGCAAGAAGCACAAGTCATGAAGAAGCTACGTCATGAGAAGCT
AGTGCAATTGTATGCAGTTGTGAGTG
>E273G
GATGCTTGGGAGATCCCTCGTGAATCACTGCGTCTTGGTGTAAAGTTAGGCCAGGGATGCTTTGG
GGAGGTGTGGATGGGCACGTGGAACGGTACTACCAGGGTTGCAATTAAGACTCTGAAACCCGGA
ACCATGTCTCCTGAGGCGTTCCTGCAAGAAGCACAAGTCATGAAGAAGCTACGTCATGAGAAGCT
AGTGCAATTGTATGCAGTTGTGAGTG
>G282A
GATGCTTGGGAGATCCCTCGTGAATCACTGCGTCTTGAGGTAAAGTTAGGCCAGGGATGCTTTGC
AGAGGTGTGGATGGGCACGTGGAACGGTACTACCAGGGTTGCAATTAAGACTCTGAAACCCGGA
ACCATGTCTCCTGAGGCGTTCCTGCAAGAAGCACAAGTCATGAAGAAGCTACGTCATGAGAAGCT
AGTGCAATTGTATGCAGTTGTGAGTG
>E283L
GATGCTTGGGAGATCCCTCGTGAATCACTGCGTCTTGAGGTAAAGTTAGGCCAGGGATGCTTTGG
GTTAGTGTGGATGGGCACGTGGAACGGTACTACCAGGGTTGCAATTAAGACTCTGAAACCCGGAA
CCATGTCTCCTGAGGCGTTCCTGCAAGAAGCACAAGTCATGAAGAAGCTACGTCATGAGAAGCTA
GTGCAATTGTATGCAGTTGTGAGTG
>G287P
GATGCTTGGGAGATCCCTCGTGAATCACTGCGTCTTGAGGTAAAGTTAGGCCAGGGATGCTTTGG
GGAGGTGTGGATGCCAACGTGGAACGGTACTACCAGGGTTGCAATTAAGACTCTGAAACCCGGA
ACCATGTCTCCTGAGGCGTTCCTGCAAGAAGCACAAGTCATGAAGAAGCTACGTCATGAGAAGCT
AGTGCAATTGTATGCAGTTGTGAGTG
>K298M
GATGCTTGGGAGATCCCTCGTGAATCACTGCGTCTTGAGGTAAAGTTAGGCCAGGGATGCTTTGG
GGAGGTGTGGATGGGCACGTGGAACGGTACTACCAGGGTTGCAATTATGACTCTGAAACCCGGA
ACCATGTCTCCTGAGGCGTTCCTGCAAGAAGCACAAGTCATGAAGAAGCTACGTCATGAGAAGCT
AGTGCAATTGTATGCAGTTGTGAGTG
>T304A
GATGCTTGGGAGATCCCTCGTGAATCACTGCGTCTTGAGGTAAAGTTAGGCCAGGGATGCTTTGG
GGAGGTGTGGATGGGCACGTGGAACGGTACTACCAGGGTTGCAATTAAGACTCTGAAACCCGGA
GCAATGTCTCCTGAGGCGTTCCTGCAAGAAGCACAAGTCATGAAGAAGCTACGTCATGAGAAGCT
AGTGCAATTGTATGCAGTTGTGAGTG
>E313R
GATGCTTGGGAGATCCCTCGTGAATCACTGCGTCTTGAGGTAAAGTTAGGCCAGGGATGCTTTGG
GGAGGTGTGGATGGGCACGTGGAACGGTACTACCAGGGTTGCAATTAAGACTCTGAAACCCGGA
ACCATGTCTCCTGAGGCGTTCCTGCAAAGAGCACAAGTCATGAAGAAGCTACGTCATGAGAAGCT
AGTGCAATTGTATGCAGTTGTGAGTG
>V316I
GATGCTTGGGAGATCCCTCGTGAATCACTGCGTCTTGAGGTAAAGTTAGGCCAGGGATGCTTTGG
GGAGGTGTGGATGGGCACGTGGAACGGTACTACCAGGGTTGCAATTAAGACTCTGAAACCCGGA
ACCATGTCTCCTGAGGCGTTCCTGCAAGAAGCACAAATTATGAAGAAGCTACGTCATGAGAAGCT
AGTGCAATTGTATGCAGTTGTGAGTG
>E323R
GATGCTTGGGAGATCCCTCGTGAATCACTGCGTCTTGAGGTAAAGTTAGGCCAGGGATGCTTTGG
GGAGGTGTGGATGGGCACGTGGAACGGTACTACCAGGGTTGCAATTAAGACTCTGAAACCCGGA

ACCATGTCTCCTGAGGCGTTCCTGCAAGAAGCACAAGTCATGAAGAAGCTACGTCATAGAAAGCT
AGTGCAATTGTATGCAGTTGTGAGTG

Block 2:
>WT
CGTCATGAGAAGCTAGTGCAATTGTATGCAGTTGTGAGTGAAGAGCCGATCTACATTGTCACTGAG
TACATGAGCAAGGGTTCTTTGCTGGACTTCTTGAAGGGTGAAACCGGCAAATACCTGAGACTTCC
CCAGTTGGTAGACATGGCCGCCCAGATTGCATCCGGTATGGCTTACGTGGAG
>L328I
CGTCATGAGAAGCTAGTGCAAATATATGCAGTTGTGAGTGAAGAGCCGATCTACATTGTCACTGAG
TACATGAGCAAGGGTTCTTTGCTGGACTTCTTGAAGGGTGAAACCGGCAAATACCTGAGACTTCC
CCAGTTGGTAGACATGGCCGCCCAGATTGCATCCGGTATGGCTTACGTGGAG
>T341I
CGTCATGAGAAGCTAGTGCAATTGTATGCAGTTGTGAGTGAAGAGCCGATCTACATTGTCATAGAG
TACATGAGCAAGGGTTCTTTGCTGGACTTCTTGAAGGGTGAAACCGGCAAATACCTGAGACTTCC
CCAGTTGGTAGACATGGCCGCCCAGATTGCATCCGGTATGGCTTACGTGGAG
>K354R
CGTCATGAGAAGCTAGTGCAATTGTATGCAGTTGTGAGTGAAGAGCCGATCTACATTGTCACTGAG
TACATGAGCAAGGGTTCTTTGCTGGACTTCTTGAGAGGTGAAACCGGCAAATACCTGAGACTTCC
CCAGTTGGTAGACATGGCCGCCCAGATTGCATCCGGTATGGCTTACGTGGAG
>D368V
CGTCATGAGAAGCTAGTGCAATTGTATGCAGTTGTGAGTGAAGAGCCGATCTACATTGTCACTGAG
TACATGAGCAAGGGTTCTTTGCTGGACTTCTTGAAGGGTGAAACCGGCAAATACCTGAGACTTCC
CCAGTTGGTAGTGATGGCCGCCCAGATTGCATCCGGTATGGCTTACGTGGAG
>A370L
CGTCATGAGAAGCTAGTGCAATTGTATGCAGTTGTGAGTGAAGAGCCGATCTACATTGTCACTGAG
TACATGAGCAAGGGTTCTTTGCTGGACTTCTTGAAGGGTGAAACCGGCAAATACCTGAGACTTCC
CCAGTTGGTAGACATGTTGGCCCAGATTGCATCCGGTATGGCTTACGTGGAG
>K354P
CGTCATGAGAAGCTAGTGCAATTGTATGCAGTTGTGAGTGAAGAGCCGATCTACATTGTCACTGAG
TACATGAGCAAGGGTTCTTTGCTGGACTTCTTGCCCGGTGAAACCGGCAAATACCTGAGACTTCC
CCAGTTGGTAGACATGGCCGCCCAGATTGCATCCGGTATGGCTTACGTGGAG
>S345P
CGTCATGAGAAGCTAGTGCAATTGTATGCAGTTGTGAGTGAAGAGCCGATCTACATTGTCACTGAG
TACATGCCAAAGGGTTCTTTGCTGGACTTCTTGAAGGGTGAAACCGGCAAATACCTGAGACTTCC
CCAGTTGGTAGACATGGCCGCCCAGATTGCATCCGGTATGGCTTACGTGGAG
>G355V
CGTCATGAGAAGCTAGTGCAATTGTATGCAGTTGTGAGTGAAGAGCCGATCTACATTGTCACTGAG
TACATGAGCAAGGGTTCTTTGCTGGACTTCTTGAAGGTCGAAACCGGCAAATACCTGAGACTTCC
CCAGTTGGTAGACATGGCCGCCCAGATTGCATCCGGTATGGCTTACGTGGAG
>M377F
CGTCATGAGAAGCTAGTGCAATTGTATGCAGTTGTGAGTGAAGAGCCGATCTACATTGTCACTGAG
TACATGAGCAAGGGTTCTTTGCTGGACTTCTTGAAGGGTGAAACCGGCAAATACCTGAGACTTCC
CCAGTTGGTAGACATGGCCGCCCAGATTGCATCCGGTTTTGCTTACGTGGAG

Block 3:
>WT
GGTATGGCTTACGTGGAGAGAATGAATTACGTACACCGTGATCTAAGAGCTGCGAACATACTGGTT
GGAGAAAACTTGGTATGTAAGGTCGCTGATTTCGGTCTGGCGAGGCTTATTGAAGACAATGAATAC
ACTGCACGTCAAGGTGCAAAGTTCCCCATCAAGTGGACGGCTCCAGAG
>E381K

GGTATGGCTTACGTGAAAAGAATGAATTACGTACACCGTGATCTAAGAGCTGCGAACATACTGGTT
GGAGAAAACTTGGTATGTAAGGTCGCTGATTTCGGTCTGGCGAGGCTTATTGAAGACAATGAATAC
ACTGCACGTCAAGGTGCAAAGTTCCCCATCAAGTGGACGGCTCCAGAG
>R382P
GGTATGGCTTACGTGGAGCCCATGAATTACGTACACCGTGATCTAAGAGCTGCGAACATACTGGTT
GGAGAAAACTTGGTATGTAAGGTCGCTGATTTCGGTCTGGCGAGGCTTATTGAAGACAATGAATAC
ACTGCACGTCAAGGTGCAAAGTTCCCCATCAAGTGGACGGCTCCAGAG
>R391A
GGTATGGCTTACGTGGAGAGAATGAATTACGTACACCGTGATCTAGCCGCTGCGAACATACTGGTT
GGAGAAAACTTGGTATGTAAGGTCGCTGATTTCGGTCTGGCGAGGCTTATTGAAGACAATGAATAC
ACTGCACGTCAAGGTGCAAAGTTCCCCATCAAGTGGACGGCTCCAGAG
>I395C
GGTATGGCTTACGTGGAGAGAATGAATTACGTACACCGTGATCTAAGAGCTGCGAACTGCCTGGT
TGGAGAAAACTTGGTATGTAAGGTCGCTGATTTCGGTCTGGCGAGGCTTATTGAAGACAATGAATA
CACTGCACGTCAAGGTGCAAAGTTCCCCATCAAGTGGACGGCTCCAGAG
>T420V
GGTATGGCTTACGTGGAGAGAATGAATTACGTACACCGTGATCTAAGAGCTGCGAACATACTGGTT
GGAGAAAACTTGGTATGTAAGGTCGCTGATTTCGGTCTGGCGAGGCTTATTGAAGACAATGAATAC
GTCGCACGTCAAGGTGCAAAGTTCCCCATCAAGTGGACGGCTCCAGAG
>R412F
GGTATGGCTTACGTGGAGAGAATGAATTACGTACACCGTGATCTAAGAGCTGCGAACATACTGGTT
GGAGAAAACTTGGTATGTAAGGTCGCTGATTTCGGTCTGGCGTTTCTTATTGAAGACAATGAATAC
ACTGCACGTCAAGGTGCAAAGTTCCCCATCAAGTGGACGGCTCCAGAG
>Y419A
GGTATGGCTTACGTGGAGAGAATGAATTACGTACACCGTGATCTAAGAGCTGCGAACATACTGGTT
GGAGAAAACTTGGTATGTAAGGTCGCTGATTTCGGTCTGGCGAGGCTTATTGAAGACAATGAAGC
AACTGCACGTCAAGGTGCAAAGTTCCCCATCAAGTGGACGGCTCCAGAG
>P434H
GGTATGGCTTACGTGGAGAGAATGAATTACGTACACCGTGATCTAAGAGCTGCGAACATACTGGTT
GGAGAAAACTTGGTATGTAAGGTCGCTGATTTCGGTCTGGCGAGGCTTATTGAAGACAATGAATAC
ACTGCACGTCAAGGTGCAAAGTTCCCCATCAAGTGGACGGCTCACGAG
>I429A
GGTATGGCTTACGTGGAGAGAATGAATTACGTACACCGTGATCTAAGAGCTGCGAACATACTGGTT
GGAGAAAACTTGGTATGTAAGGTCGCTGATTTCGGTCTGGCGAGGCTTATTGAAGACAATGAATAC
ACTGCACGTCAAGGTGCAAAGTTCCCCGCGAAGTGGACGGCTCCAGAG

Block 4:
>WT
TGGACGGCTCCAGAGGCTGCCTTATATGGAAGGTTCACGATAAAGTCCGATGTGTGGAGTTTCGG
GATATTGTTAACAGAATTGACAACGAAAGGACGTGTACCATATCCTGGCATGGTTAATAGAGAAGTA
CTTGACCAGGTAGAACGTGGTTATAGAATGCCATGCCCTCCGGAGTGTCCC
>W431V
GTCACGGCTCCAGAGGCTGCCTTATATGGAAGGTTCACGATAAAGTCCGATGTGTGGAGTTTCGG
GATATTGTTAACAGAATTGACAACGAAAGGACGTGTACCATATCCTGGCATGGTTAATAGAGAAGTA
CTTGACCAGGTAGAACGTGGTTATAGAATGCCATGCCCTCCGGAGTGTCCC
>L454A
TGGACGGCTCCAGAGGCTGCCTTATATGGAAGGTTCACGATAAAGTCCGATGTGTGGAGTTTCGG
GATAGCTTTAACAGAATTGACAACGAAAGGACGTGTACCATATCCTGGCATGGTTAATAGAGAAGT
ACTTGACCAGGTAGAACGTGGTTATAGAATGCCATGCCCTCCGGAGTGTCCC
>E473T

TGGACGGCTCCAGAGGCTGCCTTATATGGAAGGTTCACGATAAAGTCCGATGTGTGGAGTTTCGG
GATATTGTTAACAGAATTGACAACGAAAGGACGTGTACCATATCCTGGCATGGTTAATAGAACTGTA
CTTGACCAGGTAGAACGTGGTTATAGAATGCCATGCCCTCCGGAGTGTCCC
>E489S
TGGACGGCTCCAGAGGCTGCCTTATATGGAAGGTTCACGATAAAGTCCGATGTGTGGAGTTTCGG
GATATTGTTAACAGAATTGACAACGAAAGGACGTGTACCATATCCTGGCATGGTTAATAGAGAAGTA
CTTGACCAGGTAGAACGTGGTTATAGAATGCCATGCCCTCCGTCTTGTCCC
>T443W
TGGACGGCTCCAGAGGCTGCCTTATATGGAAGGTTCTGGATAAAGTCCGATGTGTGGAGTTTCGG
GATATTGTTAACAGAATTGACAACGAAAGGACGTGTACCATATCCTGGCATGGTTAATAGAGAAGTA
CTTGACCAGGTAGAACGTGGTTATAGAATGCCATGCCCTCCGGAGTGTCCC
>L458V
TGGACGGCTCCAGAGGCTGCCTTATATGGAAGGTTCACGATAAAGTCCGATGTGTGGAGTTTCGG
GATATTGTTAACAGAAGTAACAACGAAAGGACGTGTACCATATCCTGGCATGGTTAATAGAGAAGTA
CTTGACCAGGTAGAACGTGGTTATAGAATGCCATGCCCTCCGGAGTGTCCC
>T443P
TGGACGGCTCCAGAGGCTGCCTTATATGGAAGGTTCCCAATAAAGTCCGATGTGTGGAGTTTCGG
GATATTGTTAACAGAATTGACAACGAAAGGACGTGTACCATATCCTGGCATGGTTAATAGAGAAGTA
CTTGACCAGGTAGAACGTGGTTATAGAATGCCATGCCCTCCGGAGTGTCCC
>I444F
TGGACGGCTCCAGAGGCTGCCTTATATGGAAGGTTCACGTTCAAGTCCGATGTGTGGAGTTTCGG
GATATTGTTAACAGAATTGACAACGAAAGGACGTGTACCATATCCTGGCATGGTTAATAGAGAAGTA
CTTGACCAGGTAGAACGTGGTTATAGAATGCCATGCCCTCCGGAGTGTCCC
>I444P
TGGACGGCTCCAGAGGCTGCCTTATATGGAAGGTTCACGCCGAAGTCCGATGTGTGGAGTTTCG
GGATATTGTTAACAGAATTGACAACGAAAGGACGTGTACCATATCCTGGCATGGTTAATAGAGAAG
TACTTGACCAGGTAGAACGTGGTTATAGAATGCCATGCCCTCCGGAGTGTCCC

Block 5:
>WT
TGCCCTCCGGAGTGTCCCGAGAGCTTGCACGACCTTATGTGTCAGTGTTGGAGGAAAGAGCCTG
AGGAGAGGCCTACATTCGAGTATCTACAAGCATTCTTAGAAGACTACTTCACGTCCACAGAACCAC
AGTACCAACCCGGAGAGAACTTG
>D496Y
TGCCCTCCGGAGTGTCCCGAGAGCTTGCACTATCTTATGTGTCAGTGTTGGAGGAAAGAGCCTG
AGGAGAGGCCTACATTCGAGTATCTACAAGCATTCTTAGAAGACTACTTCACGTCCACAGAACCAC
AGTACCAACCCGGAGAGAACTTG
>E508G
TGCCCTCCGGAGTGTCCCGAGAGCTTGCACGACCTTATGTGTCAGTGTTGGAGGAAAGAGCCTG
AGGGCAGGCCTACATTCGAGTATCTACAAGCATTCTTAGAAGACTACTTCACGTCCACAGAACCAC
AGTACCAACCCGGAGAGAACTTG
>Y514R
TGCCCTCCGGAGTGTCCCGAGAGCTTGCACGACCTTATGTGTCAGTGTTGGAGGAAAGAGCCTG
AGGAGAGGCCTACATTCGAGAGGCTACAAGCATTCTTAGAAGACTACTTCACGTCCACAGAACCA
CAGTACCAACCCGGAGAGAACTTG
>P532W
TGCCCTCCGGAGTGTCCCGAGAGCTTGCACGACCTTATGTGTCAGTGTTGGAGGAAAGAGCCTG
AGGAGAGGCCTACATTCGAGTATCTACAAGCATTCTTAGAAGACTACTTCACGTCCACAGAACCAC
AGTACCAATGGGGAGAGAACTTG
>Y530D

TGCCCTCCGGAGTGTCCCGAGAGCTTGCACGACCTTATGTGTCAGTGTTGGAGGAAAGAGCCTG
AGGAGAGGCCTACATTCGAGTATCTACAAGCATTCTTAGAAGACTACTTCACGTCCACAGAACCAC
AGGATCAACCCGGAGAGAACTTG
>M498Q
TGCCCTCCGGAGTGTCCCGAGAGCTTGCACGACCTTCAATGTCAGTGTTGGAGGAAAGAGCCTG
AGGAGAGGCCTACATTCGAGTATCTACAAGCATTCTTAGAAGACTACTTCACGTCCACAGAACCAC
AGTACCAACCCGGAGAGAACTTG
>F523K
TGCCCTCCGGAGTGTCCCGAGAGCTTGCACGACCTTATGTGTCAGTGTTGGAGGAAAGAGCCTG
AGGAGAGGCCTACATTCGAGTATCTACAAGCATTCTTAGAAGACTACAAAACGTCCACAGAACCAC
AGTACCAACCCGGAGAGAACTTG
>Y514N
TGCCCTCCGGAGTGTCCCGAGAGCTTGCACGACCTTATGTGTCAGTGTTGGAGGAAAGAGCCTG
AGGAGAGGCCTACATTCGAGAACCTACAAGCATTCTTAGAAGACTACTTCACGTCCACAGAACCA
CAGTACCAACCCGGAGAGAACTTG
>L494P
TGCCCTCCGGAGTGTCCCGAGAGCCCACACGACCTTATGTGTCAGTGTTGGAGGAAAGAGCCTG
AGGAGAGGCCTACATTCGAGTATCTACAAGCATTCTTAGAAGACTACTTCACGTCCACAGAACCAC
AGTACCAACCCGGAGAGAACTTG

Supplementary Table 8: Kinase Atlas known allosteric pockets from the literature

Site	Site Name Origin	Inhibitor Type	Source Kinase	PDB	Pocket Description	Present in Src
DFG	DFG motif	II	many	1IEP	Hydrophobic pocket that opens up when DFG motif switches to inactive "DFG-out" conformation; binding here may stabilize inactive kinase conformation	yes
MT3	MEK1/2 type III inhibitor	III	MEK1/2	4AN2	Adjacent to ATP and DFG-out pockets; binding disrupts salt bridge required for kinase activity	yes

PIF	PDK1 interacting fragment	IV	PDK1	4RQK	PDK1 regulates other AGC kinases by recruiting them through this site	no
MPP	MKK4 p38a peptide	IV	MKK4	3ALO	p38a peptide binding inhibits MKK4 by inducing conformational changes that lead to auto-inhibition	yes
CMP	c-Abl myristoyl pocket	IV	c-Abl	3K5V	Binding here leads to active or inactive state in c-Abl (depending on ligand size) by affecting SH domain binding	yes
PMP	PKA myristoyl pocket	IV	PKA	1CMK	Myristoyl binding here activates membrane binding in PKA	no
DRS	D-recruitment site	IV	all MAPKs	1UKI	Substrate docking site present in all MAP kinases	no
DEF	docking site for ERK FXF	IV	some MAPKs	3O2M	Substrate docking site present in some MAP kinases; located near MAPK insert	no
LBP	lipid binding pocket	IV	p38a MAPK	3NEW	Binding of different lipids here affects p38a MAPK's preference and activity for different substrates	no
PDIG	PDIG motif	IV	Chk1	3JVS	Substrate recognition site located near PDIG motif in Chk1	yes
AAS	Aurora A activation segment	IV	Aurora A	4C3P	An Aurora A monomer activates another through binding of its activation segment to this site	yes
EDI	EGFR dimerization interface	IV	EGFR	2RFE	An EGFR monomer activates another by binding at this interface on the C-terminal domain	yes

Supplementary Table 9: mRNA display reagents

DNA library:
taatacgactcactatagggtctagaaataattttgtttaactttaagaaggagatatacatatgcaccatcaccaccatcatcatca
cGGATCGGGTAGTGGCATGGATGCTTGGGAGATCCCTCGTGAATCACTGCGTCTTGAGG
TAAAGTTAGGCCAGGGATGCTTTGGGGAGGTGTGGATGGGCACGTGGAACGGTACTAC
CAGGGTTGCAATTAAGACTCTGAAACCCGGAACCATGTCTCCTGAGGCGTTCCTGCAAG
AAGCACAAGTCATGAAGAAGCTACGTCATGAGAAGCTAGTGCAATTGTATGCAGTTGTGA
GTGAAGAGCCGATCTACATTGTCACTGAGTACATGAGCAAGGGTTCTTTGCTGGACTTC
TTGAAGGGTGAAACCGGCAAATACCTGAGACTTCCCCAGTTGGTAGACATGGCCGCCC
AGATTGCATCCGGTATGGCTTACGTGGAGAGAATGAATTACGTACACCGTGATCTAAGAG
CTGCGAACATACTGGTTGGAGAAAACTTGGTATGTAAGGTCGCTGATTTCGGTCTGGCG

AGGCTTATTGAAGACAATGAATACACTGCACGTCAAGGTGCAAAGTTCCCCATCAAGTG
GACGGCTCCAGAGGCTGCCTTATATGGAAGGTTCACGATAAAGTCCGATGTGTGGAGTT
TCGGGATATTGTTAACAGAATTGACAACGAAAGGACGTGTACCATATCCTGGCATGGTTA
ATAGAGAAGTACTTGACCAGGTAGAACGTGGTTATAGAATGCCATGCCCTCCGGAGTGT
CCCGAGAGCTTGCACGACCTTATGTGTCAGTGTTGGAGGAAAGAGCCTGAGGAGAGGC
CTACATTCGAGTATCTACAAGCATTCTTAGAAGACTACTTCACGTCCACAGGTAGCGGCT
CC

T7 promoter: taatacgactcactatag
RBS: gaaggag
Histag: caccatcaccaccatcatcatcac
GSGS linker: GGATCGGGTAGT
Block3:GGTATGGCTTACGTGGAGAGAATGAATTACGTACACCGTGATCTAAGAGCTGCGA
ACATACTGGTTGGAGAAAACTTGGTATGTAAGGTCGCTGATTTCGGTCTGGCGAGGCTT
ATTGAAGACAATGAATACACTGCACGTCAAGGTGCAAAGTTCCCCATCAAGTGGACGGC
TCCAGAG
GSGS linker: GGTAGCGGCTCC

Puromycin linker:
5’-/5Phos/AAA AAA AAA AAA AAA AAA AAA/iSp9//iSp9//iSp9/ACC/3Puro/-3’

Splint Oligo:
TTTTTTTTTTTTGGAGCCGCTACC
